# Global transcriptomic analysis of the arcuate nucleus following chronic glucocorticoid treatment

**DOI:** 10.1016/j.molmet.2019.05.008

**Published:** 2019-05-18

**Authors:** Jonathan R. Wray, Alison Davies, Charlotte Sefton, Tiffany-Jayne Allen, Antony Adamson, Philip Chapman, Brian Y.H. Lam, Giles S.H. Yeo, Anthony P. Coll, Erika Harno, Anne White

**Affiliations:** 1 Division of Diabetes, Endocrinology and Gastroenterology, Faculty of Biology, Medicine and Health, University of Manchester, Manchester Academic Health Sciences Centre, Manchester, M13 9PT, UK; 2 Manchester Transgenic Unit, University of Manchester, Manchester Academic Health Sciences Centre, Manchester, M13 9PT, UK; 3 Cancer Research UK Manchester Institute, Manchester, UK; 4 University of Cambridge Metabolic Research Laboratories and MRC Metabolic Diseases Unit, Wellcome-MRC Institute of Metabolic Science, Addenbrooke's Hospital, Cambridge, CB2 0QQ, UK

**Keywords:** Glucocorticoid, Arcuate nucleus, RNA-Seq, Dio2, CRISPR-Cas9

## Abstract

**Objective:**

Glucocorticoids (GCs) are widely prescribed medications that are well recognized to cause adverse metabolic effects including hyperphagia, obesity, and hyperglycemia. These effects have been recapitulated in a murine model of GC excess, and we hypothesize that they are mediated, in part, through central mechanisms. This study aimed to identify genes in the hypothalamic arcuate nucleus (ARC) that are altered with GC treatment and evaluate their contribution to GC-induced metabolic abnormalities.

**Methods:**

Corticosterone (Cort; 75 μg/ml) was administered in the drinking water to male C57Bl/6J mice for 2 days or 4 weeks. Phenotypic analysis of each group was undertaken and central and peripheral tissues were collected for biochemical and mRNA analyses. Arcuate nuclei were isolated by laser capture microdissection and tissue analyzed by RNA-seq.

**Results:**

RNA-seq analysis of ARC tissue from 4 week Cort treated mice revealed 21 upregulated and 22 downregulated genes at a time when mice had increased food intake, expansion of adipose tissue mass, and insulin resistance. In comparison, after 2 days Cort treatment, when the main phenotypic change was increased food intake, RNA-seq identified 30 upregulated and 16 downregulated genes. Within the genes altered at 2 days were a range of novel genes but also those known to be regulated by GCs, including *Fkbp5*, *Mt2*, *Fam107a*, as well as some involved in the control of energy balance, such as *Agrp*, *Sepp1*, *Dio2*, and *Nmb*. Of the candidate genes identified by RNA-seq, type-II iodothyronine deiodinase (*Dio2*) was chosen for further investigation as it was increased (2-fold) with Cort, and has been implicated in the control of energy balance via the modulation of hypothalamic thyroid hormone availability. Targeted knockdown of *Dio2* in the MBH using AAV-mediated CRISPR-Cas9 produced a mild attenuation in GC-induced brown adipose tissue weight gain, as well as a 56% reduction in the GC-induced increase in *Agrp*. However, this conferred no protection from GC-induced hyperphagia, obesity, or hyperglycemia.

**Conclusions:**

This study identified a comprehensive set of genes altered by GCs in the ARC and enabled the selection of key candidate genes. Targeted knockdown of hypothalamic *Dio2* revealed that it did not mediate the chronic GC effects on hyperphagia and hyperglycemia.

## Highlights

• RNA-seq reveals multiple genes changed by Cort in the arcuate nucleus.
• Corticosterone increases *Dio2* expression in the mediobasal hypothalamus.
• *Dio2* increase associated with greater food intake after 2 day Cort.
• CRISPR-Cas9 knockdown of *Dio2* attenuates the Cort-induced increase in *Agrp*.
• Elevated *Dio2* does not drive the GC-induced adverse metabolic effects.

## 1 Introduction

Glucocorticoids (GCs) are extensively prescribed medicines due to their potent immunosuppressive and anti-inflammatory properties. It has been estimated that, at any one time, up to 1% of the adult population in the UK and US may be receiving oral GC therapy [1], [2], [3], [4], with nearly one quarter of patients requiring GCs for longer than 6 months [2]. Prolonged high-dose GC use is associated with a range of metabolic side effects, including hyperphagia, obesity, and abnormal glucose homeostasis [4], [5], [6]. Despite their regular occurrence, the mechanisms driving these adverse metabolic effects remain unclear.

Although GCs have a variety of well-known peripheral effects, recent evidence suggests that their adverse metabolic outcomes may be mediated, at least in part, through central mechanisms. Indeed, intracerebroventricular but not peripheral infusion of the synthetic glucocorticoid receptor (GR) agonist dexamethasone (DEX) caused an increase in food intake, body weight gain, and induced insulin resistance [7], [8], [9]. A potential region involved in driving these GC-induced side effects is the hypothalamic arcuate nucleus (ARC), a region with high levels of GR expression [10] that is critical for the regulation of feeding and metabolism [11], [12], [13], [14]. The ARC contains two functionally opposed neuronal populations, expressing agouti-related peptide (AgRP), and pro-opiomelanocortin (POMC) [11], regarded as the primary energy regulatory neurons. Both neurons and glia of the ARC are responsive to peripheral hormones, including leptin, insulin, and ghrelin, which elicit many of their effects via this nucleus [15], [16], [17]. However, despite their potent orexigenic and obesogenic effects, the role of GCs in this nucleus remains relatively unexplored.

To be able to more fully investigate the central effects of chronic GCs, a model system that recapitulates the broad range of metabolic sequelae observed in humans with GC excess is required. Accordingly, a model of GC-induced metabolic syndrome has been presented by Karatsoreos *et* *al*. (2010) in which mice are delivered corticosterone (Cort) in the drinking water [18], thereby limiting the stress associated with daily injections or surgical Cort pellet implantation. In previous studies, we have characterized a similar mouse model of GC excess using Cort delivery in the drinking water [19], [20] and shown that this leads to elevated hypothalamic Cort [19]. Using this model, we have characterized the development of metabolic changes over time and reported that chronic Cort delivery successfully induces hyperphagia and obesity, as well as hyperglycemia, hyperinsulinemia, and insulin resistance [19], [20]. We also found a consistent increase in hypothalamic *Agrp* across multiple time points, leading us to investigate whether AgRP mediated the GC-induced metabolic abnormalities. However, Cort-treated *Agrp* null mice still developed obesity, hyperinsulinemia, and hyperglycemia [20], suggesting the involvement of other mechanisms.

In the current study, we used a global transcriptomic approach to investigate the effects of GCs in the hypothalamus. To impartially identify genes that are differentially expressed in the ARC with long- (4 weeks) and short-term (2 days) Cort treatment, we used our model of GC excess in combination with laser capture microdissection and RNA-seq. This revealed a variety of genes involved in energy regulation, of which type-II iodothyronine deiodinase (*Dio2*) was selected as the most promising candidate for further study. To determine the contribution of elevated *Dio2* in GC-induced hyperphagia and obesity, an AAV-mediated CRISPR-Cas9 approach was employed to knock down *Dio2* in the mediobasal hypothalamus (MBH), before mice were challenged with Cort for 4 weeks. Disruption of *Dio2* attenuated the GC-induced increase in *Agrp*; however, knockdown conferred no protection from the observed hyperphagia or weight gain.

## 2 Materials and methods

### 2.1 Animal husbandry

Male C57Bl/6J mice (10 week old; Charles River) were singly housed under 12 h light/dark cycle (lights on 7 am, zeitgeber time [ZT] 0, lights off 7 pm, ZT12) at an ambient temperature of 23 ± 1 °C with a relative humidity of approximately 40% in specific pathogen-free housing with wood chip bedding and environmental enrichment. Food (RM1-P chow; SDS) and water were available *ad libitum* throughout experiments. All experiments were performed in accordance with the UK Animals (Scientific Procedures) Act of 1986, using procedures approved by The University of Manchester Ethical Review Panel.

### 2.2 *In vivo* study design and methodology

For all studies mice were singly housed upon arrival and acclimatized for 1 week before undergoing 1–2 weeks of baseline measurements prior to study start. Food intake, water intake, and body weight were monitored either daily (2 day study) or twice weekly (4 week studies) throughout baseline and study periods. All mice were culled by rising CO_2_ (at 10 am). Tissues were snap frozen on dry ice and stored at −80 °C until use. See below for *in vivo* treatments and methodology specific to each study group.

#### 2.2.1 Mice used for RNA-seq of arcuate nuclei

Mice were assigned into two treatment groups so that each group contained mice with equivalent baseline body weight. Mice were administered either corticosterone (Cort; Sigma–Aldrich, 75 μg/ml dissolved in 1% ethanol) or vehicle (1% ethanol) in the drinking water for a period of either 2 days or 4 weeks. Mice in the 2 day group were immediately culled following treatment. At the end of the 4 week treatment period mice underwent 16 h fast (6 pm–10 am) followed by sampling for glucose and insulin. Mice were culled after being refed for 24 h and brains were taken for RNA-seq (referred to as 4 week samples henceforth).

#### 2.2.2 Mice used for Dio2 knockdown study

Mice were assigned into two groups with equivalent final baseline body weight and received intracranial injections of either AAV-gRNA or AAV-null viral particles (described below). Post-surgery, mice were monitored for 2–3 weeks to allow for recovery and expression of viral constructs. Mice then underwent a baseline period, and each group was again split into two cohorts with equivalent body weight to receive either Cort (75 μg/ml dissolved in 1% ethanol) or vehicle (1% ethanol) in the drinking water for a period of 4 weeks. This resulted in 4 treatment groups: AAV-null vehicle treated (Ctrl-Veh); AAV-null Cort treated (Ctrl-Cort); AAV-gRNA vehicle treated (KO-Veh); AAV-gRNA Cort treated (KO-Cort). A separate cohort of mice were culled 2 weeks post-injection and used to verify viral targeting by immunohistochemistry, and successful *in vivo* gene editing by droplet digital PCR (ddPCR).

#### 2.2.3 AAV vector synthesis

AAV2/8 vectors containing SaCas9 constructs were generated by UNC Vector Core (University of North Carolina, Chapel Hill, USA). Prior to packaging, constructs containing gRNA1 and gRNA2 were mixed 1:1, thus producing a heterogeneous AAV population in which 50% of the viral particles contain gRNA1 and 50% contain gRNA2 (collectively referred to as AAV-gRNA henceforth; 1.2 × 10^13^ vg/ml). A construct containing SaCas9 with no gRNA was also packaged for use as a control (AAV-null; 1.7 × 10^13^ vg/ml).

#### 2.2.4 Stereotactic surgery

Male C57Bl/6J mice at 12 weeks of age were injected with AAV viral particles while maintained under isoflurane anesthesia. Bilateral injections (100 nl/injection) were directed towards the mediobasal hypothalamus at the coordinates: AP, −1.35 mm, L ±0.3 mm, DV, −6.0 mm (from bregma). Injections were delivered using a Hamilton syringe and needle (33G).

### 2.3 Biochemical measurements

Blood samples were taken by tail-prick microsampling. Blood was either used fresh, or plasma was stored at −80 °C until use. Plasma corticosterone concentrations were quantified using an ELISA (Cayman Chemical) according to the manufacturer's instructions. All samples for the measurement of corticosterone were taken within 1 min of cage disturbance. Blood glucose was measured in fresh blood using a glucometer (Accu-Chek, Roche). Circulating insulin was quantified in fresh blood (4 week) or plasma (2 day) using an Ultrasensitive Mouse Insulin ELISA (Crystal Chem) according to the manufacturer's instructions. HOMA-IR was calculated using the formula: [glucose (mmol/l) x insulin (mU/l)]/22.5.

### 2.4 Laser capture microdissection

Brains were collected from mice after 2 days or 4 weeks of Cort treatment. Brains were snap frozen and stored at −80 °C before cryosectioning. Consecutive coronal sections (2 day, 30 μm; 4 week, 12 μm) encompassing the whole ARC (−1.46 to −2.7 mm caudal to bregma [21]) were mounted onto RNAse-free membrane (2 day; MMI) or Superfrost™ Plus slides (4 week; Thermo Scientific). Frozen sections were fixed in 95% ethanol and rehydrated in 75% and 50% ethanol. Slides were stained with cresyl violet to reveal the hypothalamic cytoarchitecture (Ambion), then dehydrated in a graded ethanol series and left to air dry prior to dissection. The entire ARC was dissected from 4 week samples using the PALM Microbeam Laser Capture Microdissection System (Zeiss), and from 2 day samples using an MMI CellCut Laser Microdissection System mounted onto an IX83 inverted microscope (Olympus). RNA was extracted using an RNeasy Micro Plus Kit (Qiagen) according to manufacturer's instructions and analyzed for RNA integrity on an Agilent 2100 Bioanalyzer using an RNA 6000 Pico Kit. All RNA that was subsequently amplified had RNA integrity (RIN) scores >6.5.

### 2.5 Library preparation, RNA-seq, and bioinformatics

2 ng of RNA was converted to cDNA using the Ovation RNA-seq System V2 (NuGEN). The resultant cDNA was then fragmented to an average size of ∼200bp using Bioruptor Pico (Diagenode) before end repair and adaptor ligation was performed using the Ovation Rapid DR Multiplex System 9–16 (NuGEN) to allow multiplex sequencing. Prepared libraries were quantified using KAPA Library Quantification Kit (KAPA Biosystems) and submitted to CRUK Cambridge institute for sequencing in a single lane on a HiSeq4000 (Illumina; 2 day samples) or HiSeq2500 (Illumina; 4 week samples). Single-end reads (50bp) were obtained with average reads of 24.67 million and 14.47 million in 2 day and 4 week samples, respectively. Read count for each sample is shown in Supplemental Table 1.

Reads were mapped to the Mus Musculus GRCm38 reference assembly (Ensembl) in RStudio using Rsubread [22] before reads to specific genes were quantified using featureCounts [23]. The average mapping rate was 74.72% for 2 day Cort treated samples and 87.35% for 4 week treated samples. Expression levels are represented as counts per million (CPM) mapped reads. Prior to differential expression analysis, global transcriptomic profiles were used to construct principal component analysis (PCA) plots to visualize the distances between samples and identify outliers (Supplemental Fig. 1). In each dataset, 2 vehicle and 1 Cort treated sample were identified as outliers and excluded from future analysis, which resulted in n = 3 vehicle and n = 4 Cort replicates for each dataset. Differential expression analysis was then performed using the DESeq2 package [24]. All genes discussed had a Benjamini-Hochberg adjusted *P* value < 0.1 (*P*adj < 0.1). RNA-seq datasets were deposited in the NCBI Gene Expression Omnibus Database (accession number GSE128148). Gene ontology enrichment analysis was performed using the PANTHER online classification tool (http://pantherdb.org/; [22]). For each dataset, upregulated and downregulated genes (*P* < 0.05, *P*adj present) were separated and used for an independent analysis, for a total of 4 analyses. Statistical overrepresentation was performed on default settings using a Fisher's Exact test with FDR multiple test correction. Upstream Regulator Analysis was performed independently on genes altered in 2 day and 4 week datasets (*P* < 0.05, *P*adj present) using Ingenuity Pathway Analysis (IPA, QIAGEN Redwood City, www.qiagen.com/ingenuity).

### 2.6 Quantification of mRNA by qRT-PCR

RNA-seq expression changes were confirmed in 2 day treated replicate samples by qRT-PCR. Briefly, cDNA was freshly prepared from total RNA (n = 2 vehicle and n = 2 Cort) used to generate the RNA-seq data as well as from 5 additional 2 day Cort, and 5 additional vehicle treated ARC samples. Gene expression was determined using a 48.48 Dynamic Array microfluidic chip on the BioMark HD Real-Time PCR System (Fluidigm). 42 differentially expressed (*P* < 0.01) genes and 5 normalization genes were quantified. Relative fold-change in expression was determined by the delta–delta Ct method (2^−ΔΔCt^) [23] using *Gapdh* as the normalizer and the vehicle group as the calibrator. The list of SYBR qRT-PCR primers used is shown in Supplemental Table 2.

### 2.7 *In situ* hybridization

*In situ* hybridization was carried out as previously described [19]. Briefly, frozen brains were coronally cryosectioned (12 μm). Representative sections through the whole hypothalamus were analyzed to ensure inclusion of all anatomical levels. *In situ* riboprobes targeting *Dio2* (NM_010050.1, 948 base pairs between 666 and 1614), and *Hr* (NM_021877.2, 840 base pairs between 1933 and 2773) were generated by gene synthesis and subcloning of the sequence (GeneArt) into pGEM-5ZF(+) (Promega) and pGEM-T Easy Vector System (Promega), respectively. The resultant plasmid constructs were linearized using appropriate restriction enzymes to generate templates for sense and antisense riboprobe synthesis using the T7 polymerase *in vitro* transcription system (Promega) in the presence of ^33^P-uridine triphosphate (PerkinElmer). Probes were hybridized overnight, and hybridization was visualized by autoradiography (Kodak Bio-Max MR film, Kodak). Films were imaged using a CoolSNAP-Pro camera (Photometrics). Signal was quantified by densitometry analysis of all autoradiograph images positive for the gene of interest.

### 2.8 Design of *Dio2* targeted guide RNAs

To generate knockdown of *Dio2* we used Clustered Regularly Interspaced Short Palindromic Repeats (CRISPR)-Cas9 technology, and designed two guide RNAs (gRNAs) in exon 1 of the Dio2 gene. The gRNA for *Staphylococcus aureus* Cas9 (SaCas9) were selected using http://crispor.tefor.net/[24]. The two gRNAs selected (PAM site in italics) were g1 = CCACGTGCTTGAGCAGAATGA-*CCGAGT* and g2 = GCCGCTCCAAGTCCACTCGCG-*GAGAGT* and were predicted to have no potential off targets with mismatches of 0, 1, 2, or 3 elsewhere in the genome, indicating their high specificity.

Oligos for the gRNAs were ligated and cloned into an all-in-one vector system (Addgene, Plasmid ID 61591 [25]) containing a CMV-driven SaCas9 cassette, with the gRNA expressed adjacent to a scaffold sequence and under the control of a ubiquitous polymerase III (U6) promoter (SaCas9-gRNA plasmid). A triple HA-epitope tag conjugated to the SaCas9 enzyme was also included for identification of SaCas9 expressing cells.

### 2.9 Cell culture, transfection, and genomic DNA isolation

Mouse embryonic fibroblast cells (NIH-3T3) were cultured in medium (DMEM, Gibco) containing 10% fetal bovine serum (Biowest). To screen for gRNA efficiency, NIH-3T3 cells were co-transfected with the SaCas9-gRNA plasmid and a plasmid containing CMV-driven GFP using FuGENE HD (Promega). 48 hr after transfection, GFP-positive cells were collected by fluorescence-activated cell sorting (FACS; CyAn ADP High-Speed Analyzer, Beckman Coulter). DNA was immediately extracted from GFP-positive cells using the PureLink Genomic DNA Mini Kit (Invitrogen) and stored at −20 °C until use.

### 2.10 Droplet digital polymerase chain reaction

For validation of *in vivo* editing, ARC tissue was dissected from injected animals using laser capture microdissection as described above (2 day samples). Hypothalamic regions to be excised were determined by the pattern of SaCas9 expression, visualized by anti-HA immunohistochemistry on adjacent slices (see below). DNA was immediately extracted using the ARCTURUS® PicoPure® DNA Extraction Kit (Applied Biosystems) and stored at −20 °C until use.

Quantification of gene editing frequency (GEF) was performed on *in vitro* (transfected 3T3 cells) and *in vivo* (laser-captured tissue) samples as described previously [26]. Prior to ddPCR, *in vivo* DNA samples underwent PCR pre-amplification (New England Biolabs) of the *Dio2* gene using the forward, 5′-ACCGAGGAGGCAGAGAAGAT-3′, and reverse, 5′-GTGAGGTCACAGCCATCGTCA-3′, primers, which flank the gRNA target sites. Reaction products were purified using a QIAquick PCR Purification Kit (Qiagen). Each ddPCR reaction mix contained: ddPCR Supermix for Probes (no dUTP; 1× Bio-Rad), forward primer, reverse primer, reference probe (FAM), InDel + probe (HEX), and gDNA template. Droplets were generated using a QX200 droplet reader (Bio-Rad) and thermal cycling was performed. Droplets were quantified using a QX200 droplet reader (Bio-Rad). DNA from SaCas9-Control treated cells was used as a negative control. All ddPCR analysis was performed with Quantasoft software (Bio-Rad) using 2D amplitude dot plots. GEF was calculated using the following formula: GEF (%) = (1-(ratio of cleaved fraction)) x 100.

All ddPCR probes were designed using Primer-BLAST (NCBI) software according to previously established guidelines [28]. All probes included a fluorophore (FAM or HEX) at the 5′ end and a black hole quencher (BHQ1) at the 3′ end. Primers and probes were as follows: forward primer, 5′-CTGATCACCCTGCAGATCCT-3’; reverse primer, 5′- CCTGTTTGTAGGCATCTAGGAG -3’; reference probe, 5′-TTCTCCAACTGCCTCTTCCTGGC-3′, InDel + probe 1, 5′-GGTCATTCTGCTCAAGCACGT-3′, InDel + probe 2, 3′-ACTCTCCGCGAGTGGACTTGGA-5’.

### 2.11 Anti-HA immunohistochemistry

Brains were coronally cryosectioned (14 μm). Sections were taken throughout the whole hypothalamus (−0.46 to −2.80 mm caudal to bregma [21]). Sections were fixed in 4% PFA, permeabilized in 0.25% Triton-X, and blocked with 10% SeaBlock (Calbiochem). For anti-HA immunohistochemistry, sections were incubated with rabbit polyclonal anti-HA (ab9110, Abcam) primary antibody (1:1000) followed by donkey anti-rabbit Alexafluor 488 (1:150) secondary antibody. Endogenous tissue autofluorescence was dampened by incubation with 0.15% Sudan Black. Slides were mounted with Prolong Gold Antifade Mountant with DAPI (Thermo Scientific). Images were collected on a M205 FA Upright Stereomicroscope (Leica) and captured using a DFC 565FX (Leica) camera through LAS AF v3.1.0.8587 software (Leica). Images were processed and analyzed using Image J.

### 2.12 Micropunch biopsies and quantification of mRNA by real-time quantitative PCR

Micropunch biopsies of the mediobasal hypothalamus were obtained from frozen AAV-injected brains. RNA was extracted using an RNeasy Micro Plus Kit (Qiagen), and cDNA was generated using a High-Capacity RNA-to-cDNA Kit (Applied Biosystems). Gene expression analysis was performed on an ABI 7900HT Fast Real-Time PCR System (Applied Biosystems) using Taqman gene expression assays and Taqman® Universal Master Mix II (Applied Biosystems). Relative quantification was performed using standard curve analysis with normalization to the reference gene *Hprt*, and nomination of the vehicle group as a calibrator. The probes used were: *Agrp*, Mm00475829_g1; *Npy*, Mm00445771_m1; *Pomc*, Mm00435874_m1; *Hprt*, Mm03024075_m1.

For all injected animals the presence of SaCas9 cDNA was determined in MBH micropunches using SYBR primers with GoTaq® qPCR Master Mix (Promega). Forward primer, 5′-CAACAAGGTGCTCGTGAAGC-3’; reverse primer 5′-CGTCTTGCTGATTCTGCCCT-3’. Samples with Ct values >33 were determined to be undetectable due to a “missed” injection, and were excluded from all future analysis.

### 2.13 Statistics

All data are represented as mean ± SEM and results were considered to be statistically significant at *P* < 0.05 unless otherwise stated. Statistical tests used were: unpaired t-test, Mann–Whitney unpaired t-test, one-way ANOVA, two-way ANOVA, or Pearson rank correlation unless otherwise stated. All statistical analysis and generation of graphs was performed using Graphpad Prism 6.0, or RStudio.

## 3 Results

### 3.1 Chronic exogenous cort treatment induces a metabolic phenotype

Prior to the RNA-seq analysis of ARC tissue, mice were given Cort in the drinking water for periods of either 4 weeks or 2 days. The phenotypic results of chronic GC treatment were consistent with our previous studies using separate cohorts of mice [19], [20]. After 4 weeks Cort treatment, there was raised circulating corticosterone levels (Supplemental Fig. 2A, Supplemental Fig. 2B), hyperphagia (Supplemental Fig. 3A, Supplemental Fig. 3B), increased weight gain (Supplemental Fig. 3C), and elevated fat mass (Supplemental Fig. 3D). Our previous study demonstrated that chronic Cort treatment produced hyperglycaemia and hyperinsulinemia in the free-fed state [20]. Here we further these findings, showing that in the fasted state, blood glucose remained unaltered (Supplemental Fig. 3E), but circulating insulin was elevated (Supplemental Fig. 3F), indicating the presence of insulin resistance (Supplemental Fig. 3G). Phenotypic analysis of the 2 day cohort revealed an increase in food intake (Supplemental Fig. 4A) and brown adipose tissue mass, with no change in body weight, white adipose tissue, or gastrocnemius muscle mass (Supplemental Fig. 4B, Supplemental Fig. 4C). Additionally, blood glucose was reduced (Supplemental Fig. 4D) and insulin was increased (Supplemental Fig. 4E).

### 3.2 Global transcriptomic response in the ARC to chronic cort treatment

**Figure 1:**
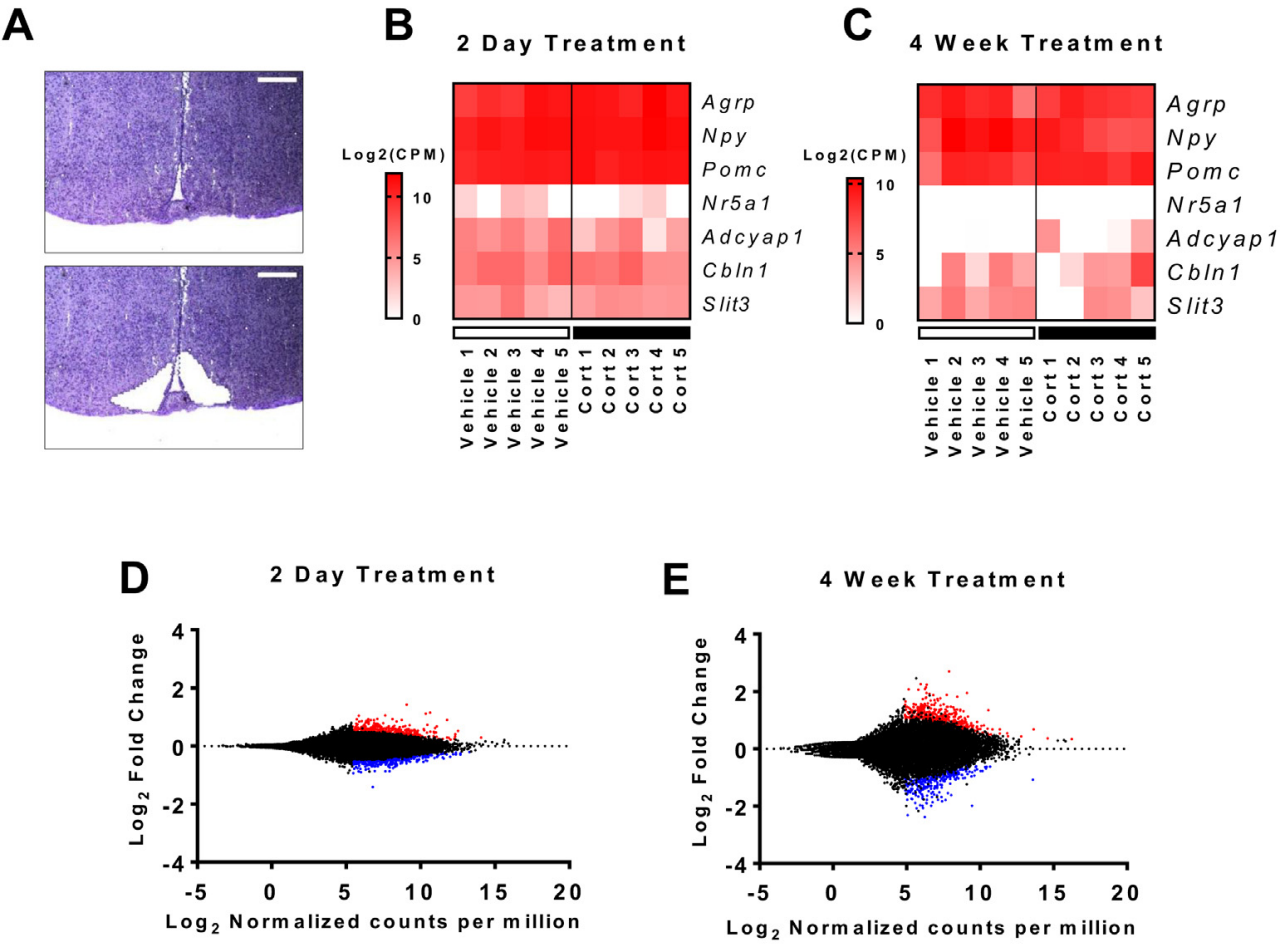
**Validation of RNA-seq datasets from the arcuate nuclei of 2 day and 4 week Cort treated mice. (A)** Representative hypothalamic sections stained with cresyl violet to allow visualization of hypothalamic cytoarchitecture and isolation of the ARC by laser capture microdissection. Scale bar = 300 μm. **(B)** Heatmap showing ARC and VMH enriched genes across all 2 day (n = 5/group) and **(C)** 4 week (n = 5/group) treated samples. **(D)** Scatter plot showing the expression and fold change of all genes in 2 day (n = 3–4/group), and **(E)** 4 week treated (n = 3–4/group) datasets. (D, E) Red dots, upregulated genes (*P* < 0.01); blue dots, downregulated genes (*P* < 0.01). Wald test.

To determine the transcriptomic response of the ARC to GC treatment, we utilized laser capture microdissection (Figure 1A) in conjunction with RNA-seq. The specificity of dissections was confirmed by analyzing genes enriched in the ARC, and in the adjacent ventromedial hypothalamus (VMH). The robust expression of *Agrp*, *Npy*, and *Pomc* was confirmed in all ARC replicates (Figure 1B,C), which showed minimal expression of the VMH enriched genes *Adcyap1*, *Cbln1*, and *Slit3* (Figure 1B,C). Expression of the specific VMH marker gene, steroidogenic factor 1 (*Nr5a1*) was absent from all 4 week replicates (Figure 1C) and expressed at >550-fold lower than *Agrp*, *Npy*, or *Pomc* in all 2 day replicates (Figure 1B), indicating a high level of accuracy when dissecting arcuate nuclei. After 4 weeks of GC treatment, the magnitude of gene changes in the ARC was greater than at 2 days, which is most clearly seen when the data are represented as scatter plots (Figure 1D,E).

### 3.3 Transcriptomic changes in the ARC induced by 4 week chronic cort treatment

**Figure 2:**
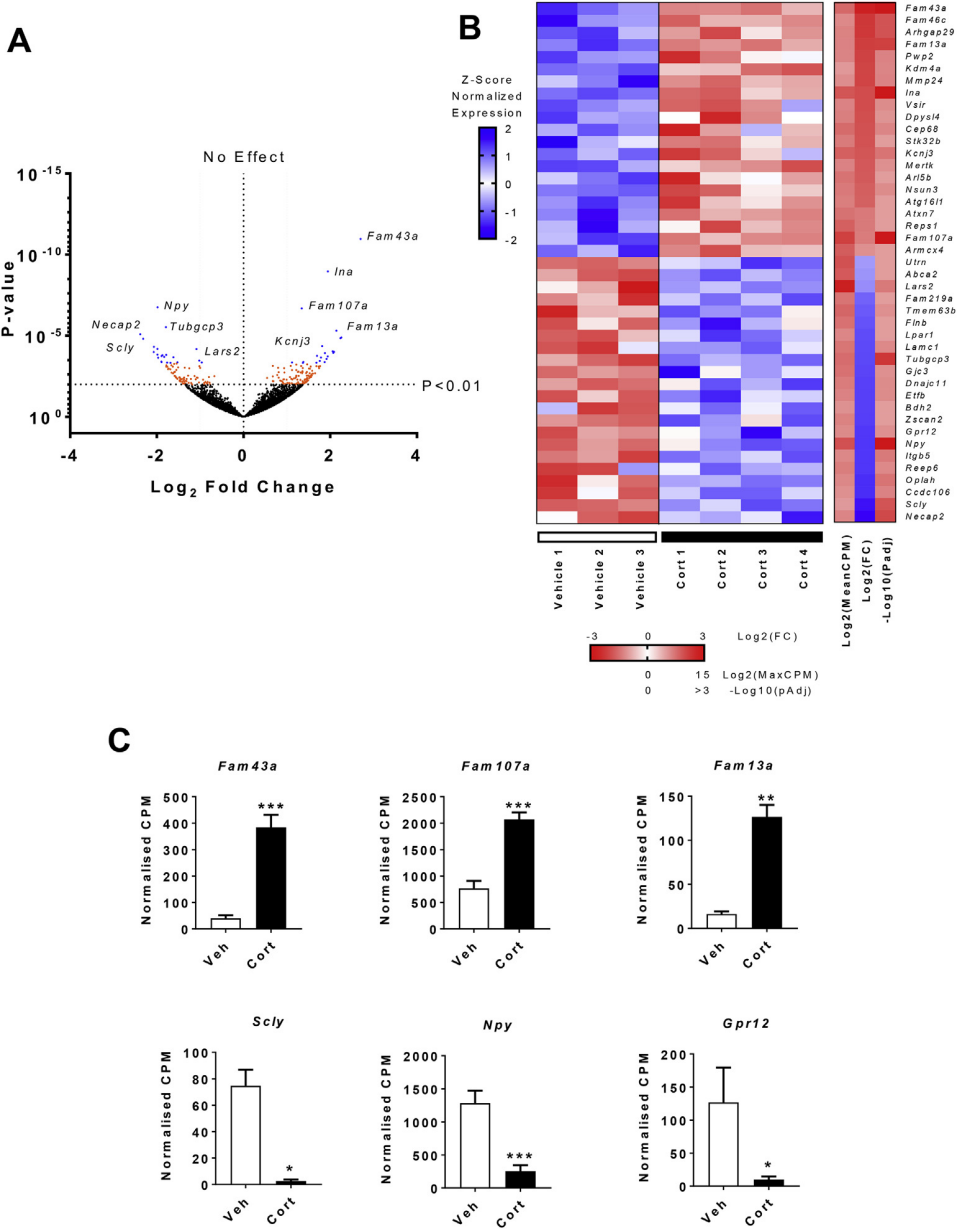
**Global gene expression changes in the arcuate nucleus of 4 week Cort treated mice. (A)** Volcano plot of all genes found by RNA-seq. Orange (*P* < 0.01) and blue (*Padj* < 0.1) dots represent genes with significant change in expression. The position of select genes is illustrated by the gene symbol (n = 3–4/group). **(B)** Heatmap showing all 43 genes differentially expressed (*Padj*) in the ARC after 4 weeks of Cort treatment. Left panel, each row corresponds to transcripts where the expression is normalized to the mean expression on the row (Z-scaled). Right panel, the mean expression across all samples [Log2(MeanCPM)], fold change [Log2(FC)], and significance for each gene [-Log10(*Padj*)] (n = 3–4/group). **(C)** Examples of different genes altered in the ARC with Cort treatment. White bars, vehicle (Veh); black bars, 75 μg/ml corticosterone (Cort) treated (n = 3–4/group; *Fam43a*, *P*adj = 9.54e-8; *Fam107a*, *P*adj = 4.63e-4; *Fam13a*, *P*adj = 0.0073; *Scly*, *P*adj = 0.014; *Npy*, *P*adj = 1.75e-7; *Gpr12*, *P*adj = 0.071). *Padj < 0.1, **Padj < 0.01, ***Padj < 0.001. (A) Wald test, (B, C) Benjamini-Hochberg adjusted *P* value.

In 4 week treated samples, 15,719 genes were expressed in the ARC (>1 normalized CPM), of which 12,013 were expressed in all samples. The number of genes differentially expressed with chronic Cort treatment was 224 (*P* < 0.01; Figure 2A, orange and blue dots), which was reduced to 43 when the data was corrected for multiple comparisons testing (*Padj* < 0.1; Figure 2A, blue dots). From these genes, 21 were upregulated, and 22 were downregulated by Cort (Figure 2B). Some of the most significantly altered genes are shown in Figure 2C. Among those upregulated are a number of genes known to be regulated by GCs including *Fam43a*, *Fam107a, Fam13a*, and *Mertk* [27]. Downregulated genes included those involved in the regulation of energy balance, including *Scly* and *Gpr12* [28], [29]. In contrast to our previous work [19], [20], *Npy* was markedly reduced and no increase in *Agrp* was observed, highlighting the issue that the fasting/refeeding (included to determine if glucose homeostasis was abnormal) has affected part of this pathway. We therefore focused on a new cohort of mice in which RNA-seq was performed on the ARC after 2 days of Cort treatment, when secondary effects, such as increased body weight, elevated fat mass, and chronic hyperinsulinemia, were limited.

Gene ontology enrichment analysis was also performed to identify groups of functionally related genes. All significant (*P*adj < 0.05) ontology terms for up- and downregulated genes at 4 weeks are detailed in Supplemental Tables 3 and 4. Within the upregulated genes there was enrichment for those involved in metabolic processes, metal ion binding, and voltage gated potassium channel activity. Downregulated genes were enriched for those involved in cellular metabolic processes, and nervous system development. Among the downregulated genes, there was an overrepresentation of several pathways known to be regulated by GCs, including neurodevelopment, axon guidance, metabolism of amino acids, and regulation of the pro-inflammatory transcription factor NfKB. Among the predicted upstream regulators (Supplemental Table 7) were those known to be regulated by GR, including heat shock factor 1 (HSF1) and amyloid precursor protein (APP), as well as those involved in the control of feeding, such as forkhead box O3 (FOXO3) and FEV (also known as PET-1).

### 3.4 Transcriptomic changes in the ARC after 2 days of cort treatment

**Figure 3:**
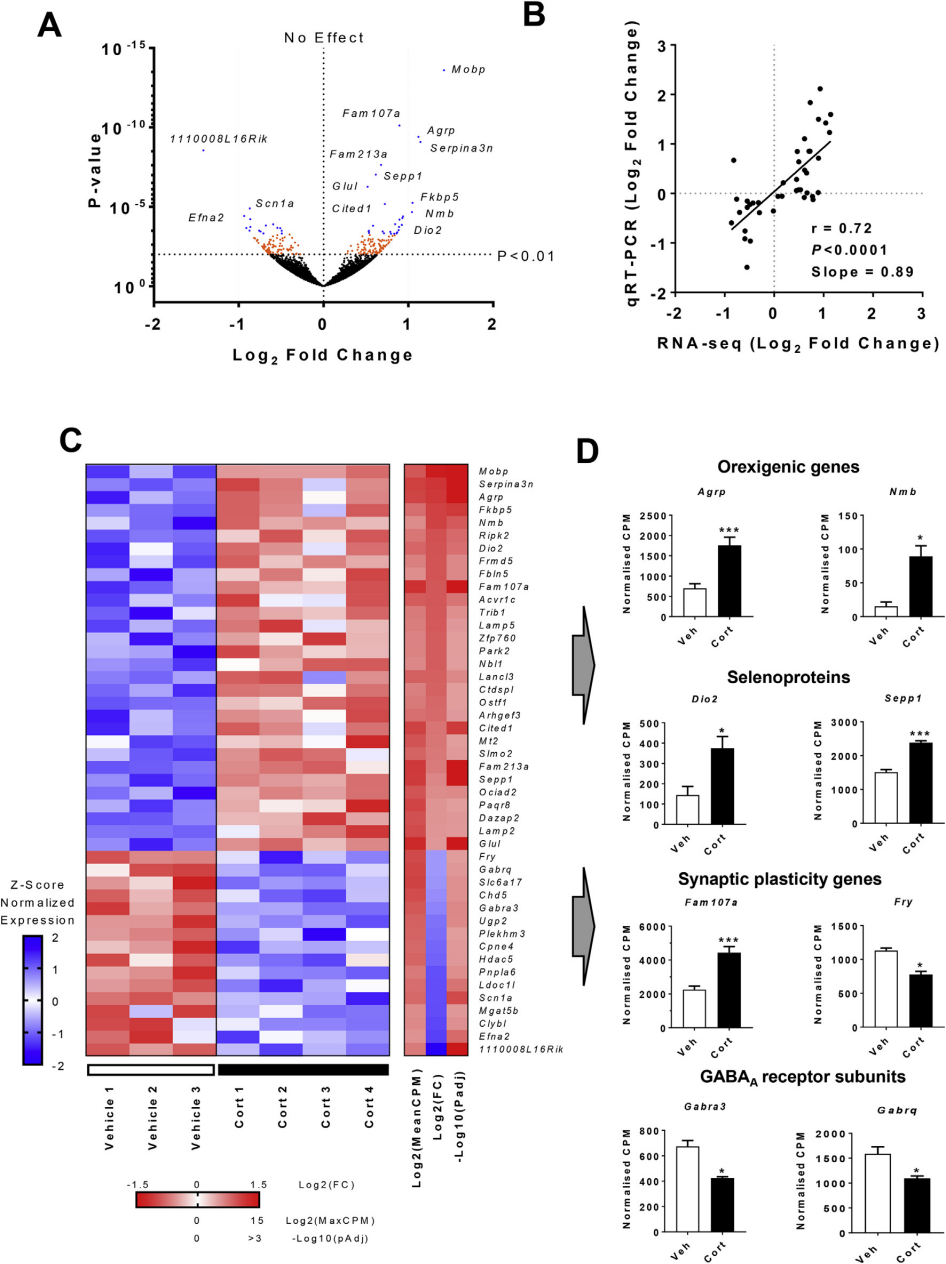
Global gene expression changes in the arcuate nucleus of 2 day Cort treated mice. **(A)** Volcano plot of all genes found by RNA-seq. Orange (*P* < 0.01) and blue (*Padj* < 0.1) dots represent genes with significant change in expression. The position of select genes is illustrated by the gene symbol (n = 3–4/group). **(B)** Biological and technical correlation between RNA-seq and qRT-PCR in 37 differentially expressed genes (RNA-seq, n = 3–4/group; qRT-PCR, n = 6–7/group). **(C)** Heatmap showing all 46 genes differentially expressed (*Padj*) in the ARC after 2 days of Cort treatment. Left panel, each row corresponds to transcripts where the expression is normalized to the mean expression on the row (Z-scaled). Right panel, the mean expression across all samples [Log2(MeanCPM)], fold change [Log2(FC)], and significance for each gene [-Log10(*Padj*)] (n = 3–4/group). **(D)** Examples of genes altered in the ARC with Cort treatment (n = 3–4/group; *Agrp*, *P*adj = 1.21e-6; *Nmb*, *P*adj = 0.016; *Dio2*, *P*adj = 0.031; *Sepp1*, *P*adj = 1.24e-4; *Fam107a*, *P*adj = 3.52e-7; *Fry*, *P*adj = 0.0995; *Gabra3*, *P*adj = 0.061; *Gabrq*, *P*adj = 0.089). White bars, vehicle (Veh); black bars, 75 μg/ml corticosterone (Cort) treated. *Padj < 0.1, **Padj < 0.01, ***Padj < 0.001. (A) Wald test, (B) Pearson's correlation, (C, D) Benjamini-Hochberg adjusted *P* value.

After 2 days of Cort treatment, 231 genes were altered in the ARC (*P* < 0.01; Figure 3A, orange and blue dots), which was reduced to 46 when corrected for multiple testing (*Padj* < 0.1; Figure 3A, blue dots). Of these genes, 30 were upregulated, and 16 were downregulated with Cort treatment. To confirm these results, a selection of significant genes (*P* < 0.01) were validated by qRT-PCR using laser captured ARC tissue from 2 day Cort treated mice. There was a clear correlation between expression data obtained using RNA-seq, and qRT-PCR (Figure 3B; Supplemental Fig. 5), confirming the 2 day RNA-seq results. All of the transcripts that were altered in the ARC after 2 days of Cort treatment are shown in Figure 3C, ranked according to their fold change. Among the upregulated genes were a number of known GC-regulated genes, such as *Fam107a*, *Fkbp5*, *Glul*, *Mt2*, *Ociad2,* and *Paqr8* [27]. A selection of the most significantly altered genes is shown in Figure 3D. Among these are the orexigenic neuropeptides *Agrp* and *Nmb* and the selenoproteins *Dio2* and *Sepp1*.

Gene ontology enrichment analysis of upregulated genes showed an over-representation of those involved in hormone secretion, with an enrichment of genes localized at the neuronal synapse (Supplemental Table 5). Within the downregulated genes there was an enrichment of those involved in neural development, synaptic transmission, calcium-dependent phospholipid binding, and locomotor activity (Supplemental Table 6). Similarly, the pathways altered included transmission across a chemical synapse and transmembrane transport of small molecules, suggesting altered neuronal activity. Upstream Regulator Analysis indicated that GR (*Nr3c1*) was among the top predicted higher order transcriptional regulators within the dataset (Supplemental Table 8), which also included APP and FEV/PET1, and these were common to the 4 week dataset.

### 3.5 Cort treated mice show increased *Dio2* expression in the ARC

**Figure 4:**
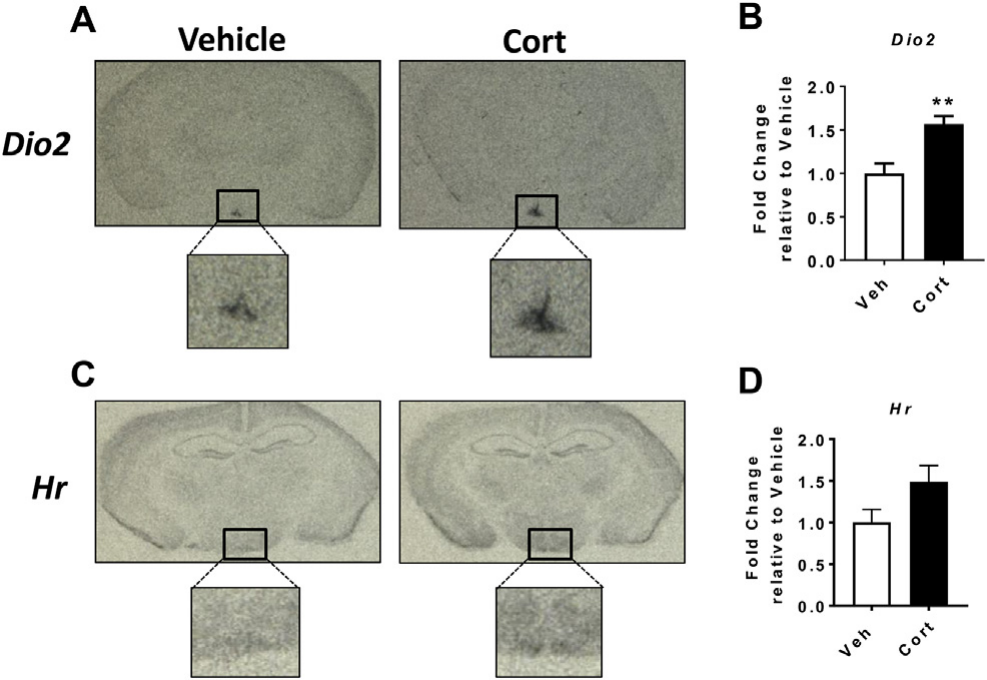
Cort treatment increases *Dio2* mRNA expression in the mediobasal hypothalamus. Mice were treated with either corticosterone or vehicle in the drinking water for 2 days. **(A, C)** Representative autoradiographic images and **(B, D)** densitometric quantification of coronal brain sections incubated with riboprobes targeting *Dio2* and *Hr,* respectively (n = 6/group; *Dio2*: *P* = 0.0022; *Hr*: *P* = 0.0931). White bars, vehicle (Veh); black bars, 75 μg/ml corticosterone (Cort) treated. **P < 0.01. (B, D) Mann–Whitney test.

An analysis of the available literature highlighted *Dio2* as an interesting candidate gene for further study, since it increases the local availability of triiodothyronine (T3), which, in turn, increases food intake. Consistent with RNA-seq and qRT-PCR, mRNA expression of *Dio2* in the MBH was increased after 2 days of Cort treatment when quantified by *in situ* hybridization (Figure 4A,B). *Dio2* expression appeared to be increased in both the tanycytes of the third ventricle and in the ARC (Figure 4A,B). Elevated *Dio2* (∼1.6-fold) was also observed in the hypothalami of mice treated with Cort for 3 weeks using the same paradigm (Supplemental Fig. 6; using 3 week hypothalamic RNA taken from Sefton et al., 2019 [20]), suggesting that the increase is sustained for the duration of Cort treatment. Furthermore, mRNA expression of the T3 responsive gene *Hr* [30] showed a trend towards an increase after 2 days of Cort (Figure 4C,D).

### 3.6 *Dio2* knockdown in the mediobasal hypothalamus using AAV-mediated CRISPR-Cas9

**Figure 5:**
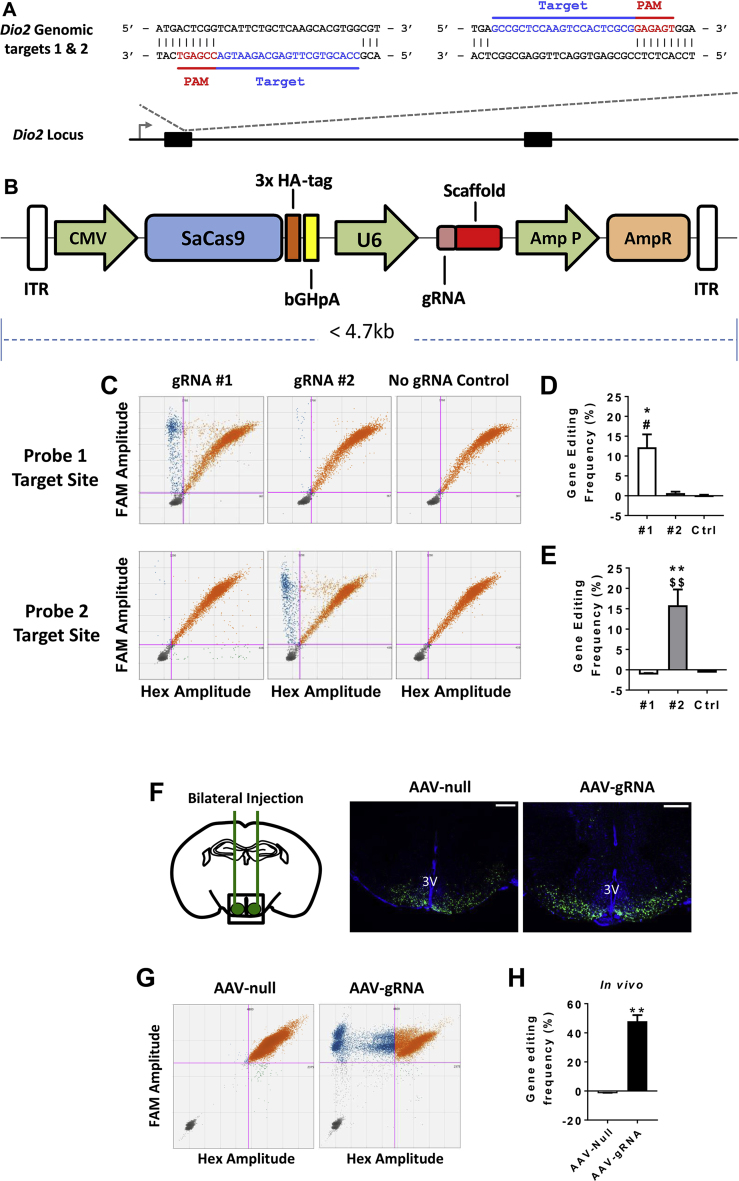
Validation of AAV-gRNA for targeted disruption of *Dio2* in the mediobasal hypothalamus. **(A)** Schematic representation of the *Dio2* locus showing the location of the gRNA target sequences. Target sequence in blue, protospacer adjacent motif sequence in red. **(B)** Schema of the single vector AAV delivery construct containing SaCas9 and gRNA**. (C)***In vitro* confirmation of gRNA efficiency using droplet digital PCR (ddPCR; n = 3–4/group). **(D)** Quantification of gRNA1 (#1) editing frequency (n = 3–4/group; F_2, 7_ = 9.057, *P* = 0.0114). **(E)** Quantification of gRNA2 (#2) editing efficiency (n = 3–4/group; F_2, 7_ = 21.62, *P* = 0.001). **(F)** Representative immunofluorescent images showing bilateral targeting of the MBH. Scale bar = 500 μm. **(G)** Confirmation and **(H)** quantification of *in vivo* AAV-gRNA gene editing efficiency (n = 4–6/group; *P* = 0.0095). All ddPCR scatter plots represent merged replicates. Reference probe, FAM; InDel + probe, Hex. Pink bars indicate manually set thresholds. Orange dots, wild type DNA; blue dots, DNA containing InDels; grey dots; empty droplets. *P < 0.05, **P < 0.01 vs Ctrl (no gRNA). ^#^*P* < 0.05 vs gRNA2, ^$$^*P* < 0.01 vs gRNA1, ***P < *0.001*. (D, E) One-way ANOVA with Tukey's post-hoc comparison, (H) Mann–Whitney test.

To investigate the role of elevated *Dio2* in the development of GC-induced metabolic effects, we next sought to knock down *Dio2* in the MBH using AAV-mediated CRISPR-Cas9. First, two gRNAs were designed to target exon 1 of the *Dio2* gene (Figure 5A) and individually inserted into an SaCas9 vector (Figure 5B). The efficiency of gRNAs at generating insertion/deletion (InDel) mutations in the *Dio2* gene was then assessed *in vitro* by transfection of the plasmid constructs into a murine embryonic fibroblast (3T3) cell line. Wild type amplicons (orange dots) and DNA containing InDels (blue dots) were detected using ddPCR with probes designed over the gRNA cut sites (Figure 5C), revealing gRNA efficiencies of 12.2% (gRNA1; Figure 5D) and 15.8% (gRNA2; Figure 5E).

Vector constructs were then packaged into AAV2/8 virus particles, a serotype that efficiently transduces mouse glial cells [31], including astrocytes and tanycytes of the ARC [12]. In order to maximize knockdown efficiency both gRNAs were packaged and used in tandem. To generate knockdown of *Dio2 in vivo*, AAV-gRNA virus particles were stereotactically injected into the MBH of wild type C57Bl/6J mice. Successful bilateral injection was confirmed by the localized expression of SaCas9, as evidenced by anti-HA immunofluorescent staining (Figure 5F). Confirmation of protein knockdown was prevented due to the lack of an anti-DIO2 antibody with suitable specificity. Similarly, CRISPR-edited alleles will still produce a non-functional mRNA transcript, making transcriptomic analysis of *Dio2* not viable as an option for knockdown confirmation. However, the presence of InDels in DNA from laser captured MBH tissue was confirmed using ddPCR (Figure 5G,H), indicating that AAV-gRNA viral particles are able to generate InDel mutations *in vivo* with high efficiency (∼50%). Individual plots for each animal can be viewed in Supplemental Fig. 7.

### 3.7 Determining if elevated *Dio2* mediates GC-induced obesity and hyperphagia

Using this model of *Dio2* knockdown, we sought to determine the contribution of elevated *Dio2* in the development of GC-induced hyperphagia and obesity. Accordingly, a separate cohort of mice were each injected with AAV-gRNA or AAV-null viral particles into the MBH and were subsequently challenged with Cort over 4 weeks. To confirm correct targeting of the virus, SaCas9 mRNA was quantified in punch biopsies taken of the MBH (Supplemental Fig. 8). Samples with absent expression (Ct > 33) were determined to be “missed” injections and were excluded from all further analysis (1 Ctrl-Veh, 1 Ctrl-Cort, 2 KO-Cort).

**Figure 6:**
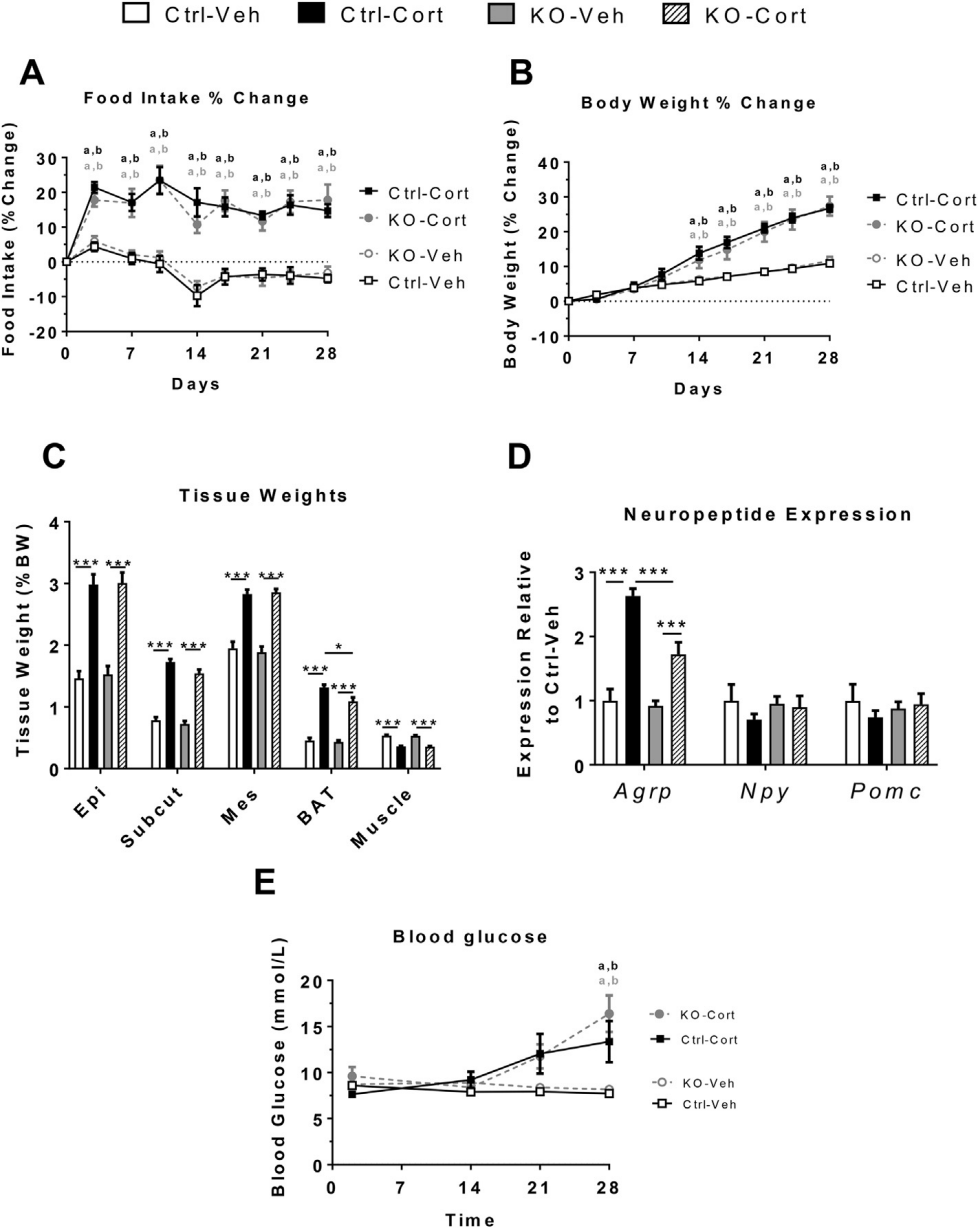
**Targeted knockdown of *Dio2* in the MBH does not alleviate the metabolic effects of chronic Cort treatment. (A)** Percent change in food intake (n = 9–14/group; time: F_8, 320_ = 19.39, *P* < 0.0001; interaction: F_24, 320_ = 5.466, *P* < 0.0001; treatment: F_3, 40_ = 34.52, *P* < 0.0001) and **(B)** body weight across the 4 week Cort treatment period (n = 9–14/group; time: F_8, 320_ = 268.6, *P* < 0.0001; interaction: F_24, 320_ = 21.23, *P* < 0.0001; treatment: F_3, 40_ = 10.52, *P* < 0.0001). **(C)** Individual adipose tissue bed and muscle weight after 4 weeks of Cort treatment (n = 8–14/group; Epi: F_3, 38_ = 31.22, *P* < 0.0001; Subcut: F_3, 39_ = 85.87, *P* < 0.0001; Mes: F_3, 38_ = 39.67, *P* < 0.0001; BAT: F_3, 39_ = 88.7, *P* < 0.0001; Muscle: F_3, 39_ = 1.945, *P* = 0.1382). **(D)** Expression of ARC neuropeptides in MBH micropunches after 4 weeks of Cort treatment (n = 8–14/group; *Agrp*: F_3, 37_ = 26.69, *P* < 0.0001; *Npy*: F_3, 38_ = 0.4874, *P* = 0.6931; *Pomc*: F_3, 37_ = 0.379, *P* = 0.7687). **(E)** Blood glucose measurements (n = 9–14/group; time: F_3, 120_ = 10.7, *P* < 0.0001; interaction: F_9, 120_ = 6.501, *P* < 0.0001; treatment: F_3, 40_ = 4.992, *P* = 0.0049). Epi, epididymal; Subcut, subcutaneous; Mes, mesenteric. Ctrl, AAV-Null injected; KO; AAV-gRNA injected; Veh, 1% ethanol; Cort, 75 μg/ml corticosterone. a, significance vs Ctrl-Veh; b, significance vs KO-Veh. *P < 0.05, **P < 0.01, ***P < 0.001. (A, B, E) Two-way ANOVA with Tukey's post-hoc compassion (C, D) one-way ANOVA with Tukey's post-hoc comparison.

In agreement with previous data, exogenous Cort treatment increased food intake (Figure 6A) and body weight (Figure 6B); however, this effect was not attenuated with *Dio2* knockdown (Figure 6A,B). The increased body weight was likely due to increased fat mass as epididymal, subcutaneous, mesenteric, and brown adipose tissues were increased, while muscle weight decreased (Figure 6C). Interestingly, the GC-induced increase in BAT weight was mildly attenuated with *Dio2* knockdown (Figure 6C). Consistent with 2 day RNA-seq data, *Agrp* mRNA expression was increased with Cort treatment in both control and *Dio2* knockdown mice, however the amplitude of this increase was reduced by 56% with *Dio2* knockdown (Figure 6D). In agreement with our previous work [19], [20], expression of *Npy* and *Pomc* was unaffected by chronic Cort treatment, and *Dio2* knockdown had no additional effect (Figure 6D). Chronic Cort treatment led to an increase in blood glucose after 28 days, measured in the free-fed state, which was not attenuated with knockdown of *Dio2* (Figure 6E).

## 4 Discussion

Long-term GC treatment was associated with the development of hyperphagia, obesity, and insulin resistance, as we have previously shown in other studies using this model [19], [20]. We hypothesized that GC action within the ARC would contribute to these effects. However, targeting specific genes has not yet identified the contributing mechanisms [20]. Therefore, in the current study, we explored the underlying mechanisms using a broad approach and performed global transcriptomic analysis of the ARC, a valuable tool allowing for the accurate and unbiased identification of genes differentially regulated between treatments. Using this approach, we have identified ARC genes altered in response to both short- (2 day) and long-term (4 week) exogenous Cort treatment and have suggested both known and novel candidate genes that merit further investigation.

In the current study, we identified 46 genes significantly altered in the ARC after 2 days of Cort treatment. Among these genes are previously identified GC target genes, including *Fam107a* and *Fkbp5*. *Fam107a* (also known as *Tu3a* and *Drr1*), is increased by both DEX [32] and prednisolone [33] and is reliant upon GR dimerization for its transactivation [33]. Similarly, *Fkbp5* is a well-recognized GC target gene [34] and increased expression in the hypothalamus has been linked to obese phenotypes [35]. The presence of known GC-sensitive genes within the dataset validates the GC delivery method used and demonstrates that the ARC is transcriptionally sensitive to circulating GCs.

In the 4 week dataset, 43 genes were significantly altered in the ARC. Surprisingly, only one gene, *Fam107a,* was altered in both 2 day and 4 week datasets. This may indicate that different mechanisms are active in the early and late stages of chronic Cort treatment. Indeed, this has been observed in another study of GC-regulated gene expression, showing that expression profiles differ based on the duration of GC exposure [36]. However, it is also likely that expression in the 4 week dataset has been influenced by the fast/refeed period, making comparisons between datasets difficult to interpret. Interestingly, FEV, a member of the highly conserved ETS family of transcription factors was a predicted upstream regulator in both 2 day and 4 week datasets. FEV/PET1 is a key regulator of serotonergic neuronal development in the CNS [37], and has recently been identified as having a key role in food-related behavioral state switching and satiety [38]. This may represent a novel pathway through which GCs regulate food intake in the ARC.

In addition to identifying well-established GC-responsive genes, we observed a change in a range of energy regulatory genes. There was a strong increase in *Nmb*, a bombesin-related peptide that potently activates AgRP/NPY neurons in the ARC [39] and may contribute to the increase in food intake. Previous studies indicate that *Nmb* is negatively regulated in the ARC by the anorexigenic hormone leptin [40] and thus may represent a mechanism mediating the bidirectional control of food intake in response to peripheral hormonal cues. Other notable genes that were downregulated with Cort treatment are *Gabra3* and *Gabrq*, two subunits of the GABA_A_ receptor. Within the ARC, GABA receptors have been implicated in several critical homeostatic mechanisms, including food intake, thermoregulation, and foraging [41]. We also observed an increase in the selenoprotein, *Sepp1*, a class of protein increasingly believed to play a key role in hypothalamic control of energy balance [42]. Indeed, elevated serum selenoprotein-P (encoded by *Sepp1*) has been associated with obesity and diabetes in humans, and overexpression both *in vitro* and *in vivo* leads to insulin resistance [43].

Among the transcripts altered in response to Cort treatment, *Dio2* stood out, both by its degree of upregulation, its restricted localization to the MBH [44], and by the growing body of evidence implicating *Dio2* and T3 in the central control of energy balance [45], [46], [47], [48], [49]. While previous studies have hinted that GCs may regulate *Dio2* [50], here we provide the first conclusive evidence that exogenous Cort increases hypothalamic *Dio2* expression. Within the hypothalamus, *Dio2* is highly localized to the tanycytes and astrocytes of the ARC [44] and increases the local availability of T3 [51], which then regulates neuronal gene expression in a paracrine manner [52]. Modulation of hypothalamic T3 can have a significant impact on food intake and metabolism. Endogenously elevated T3 acts as a key seasonal switch between catabolic and anabolic states in natural animal models of obesity, such as the Siberian hamster [45], [46]. Similarly, in other rodent models, elevated hypothalamic T3 leads to an increase in food intake [47], [48], [49]. Specifically within the ARC, T3 increases food intake via two independent mechanisms. Firstly, in fasted mice DIO2-derived T3 induces mitochondrial upregulation of UCP2 in AgRP/NPY neurons, thereby elevating their activity and increasing food intake [47]. Secondly, in the context of hyperthyroidism, T3 increases mTOR signaling in the ARC, leading to an increase in food intake [49]. It must be noted that in another study, intra-ARC injection of T3 did not alter food intake [53]. Thus while the appetitive action of hypothalamic T3 has been identified under specific circumstances, the full range of molecular mechanisms governing T3 control of food intake have yet to be elucidated.

To investigate the effects of *Dio2* on Cort-induced metabolic dysfunction, we used AAV-targeted CRISPR-Cas9 to specifically knockdown *Dio2* in the MBH before treating with Cort for 4 weeks. This knockdown of *Dio2* did not attenuate GC-induced hyperphagia or body weight gain as expected. One possibility is that the absence of a change in food intake is due to the duration of GC treatment. To date, studies that have observed elevated T3-induced food intake have done so in an acute setting. A single bolus injection of T3 into the ARC increased food intake over 12 h [49]. Similarly, global knockout of *Dio2* abolishes the fasting-induced increase in hypothalamic T3 levels, which is associated with a blunted refeeding response, only in the first 30 min after a 24 h fast [47]. Therefore, while elevated T3 increases food intake in an acute setting, this may not occur with chronic elevation. Indeed, differing acute and chronic effects of T3 were observed in other hypothalamic nuclei, as acute [54] but not chronic [55], delivery of T3 into the PVN raised blood glucose levels.

In accordance with our previous data [19], [20], GC treatment in the Cort-treated mice induced a strong increase in hypothalamic *Agrp.* This increase was attenuated (by 56%) in mice with *Dio2* knockdown, suggesting that the GC-induced increase in *Agrp* is mediated, at least partially, by increased hypothalamic *Dio2* expression. Recent work from our lab has shown that *Agrp* deletion had little effect on Cort induced metabolic abnormalities [20], and as such this reduction in AgRP would not be expected to have a metabolic effect. The attenuated increase in *Agrp* transcript with *Dio2* knockdown is in agreement with previous evidence indicating that thyroid hormones increase the expression of *Agrp* [49], [56], [57]. It would therefore be expected that *Dio2* knockdown would decrease T3 availability, and could thus be the mechanism limiting the GC-induced increase in *Agrp.* We cannot exclude the possibility that this decrease is only partial due to incomplete knockdown of *Dio2* (50% editing efficiency); however, it may also suggest that GCs regulate *Agrp* expression through multiple mechanisms. Indeed, there is a GC response element (GRE) in the *Agrp* gene [58]. Therefore, it is likely that GCs increase *Agrp* both via direct action, and indirectly via a *Dio2*-mediated increase in local T3 availability. This hypothesis is supported by evidence that DIO2-producing glial cells are in direct contact with AgRP/NPY neurons [47], exposing them to locally formed T3.

## 5 Conclusion

This genome-wide analysis has identified the transcriptomic response of the hypothalamic ARC to chronic exogenous Cort treatment. The range of both known and novel candidate genes indicates that there is the potential for multiple mechanisms to contribute to the metabolic abnormalities observed with Cort treatment. *Dio2* was an obvious candidate given its robust elevation with Cort treatment and its clear links to feeding and metabolism. However, knockdown of *Dio2* using AAV-mediated CRISPR-Cas9 vectors targeted to the MBH conferred no protection from GC-induced hyperphagia or obesity in this setting, providing evidence that other mechanisms mediate the metabolic sequelae resulting from chronic GC treatment.

## Author contributions

JRW collected, analyzed, and interpreted the data, and drafted the article. AD was involved in the design of the experiments. AD, CS, and TA were involved with data collection and provided support throughout the course of the study. AA assisted with design of CRISPR experiments and provided expertise in this regard. BYHL and GSHY assisted with the design and execution of the RNA-seq experiments. PC assisted with bioinformatics analysis of RNA-seq data. APC, EH, and AW conceived and contributed to the design of the study. JRW, EH, and AW wrote and revised the manuscript and were responsible for its final content. All authors have approved the final version of the manuscript.
