# Supplementary material for: Global transcriptomic analysis of the arcuate nucleus following chronic glucocorticoid treatment
Source: Mol Metab. 2019 May 18;26:5–17. doi: 10.1016/j.molmet.2019.05.008 (PMC6667392; doi:10.1016/j.molmet.2019.05.008)
Supplement: Multimedia component 2 [file mmc2.pptx]

## Slide 1
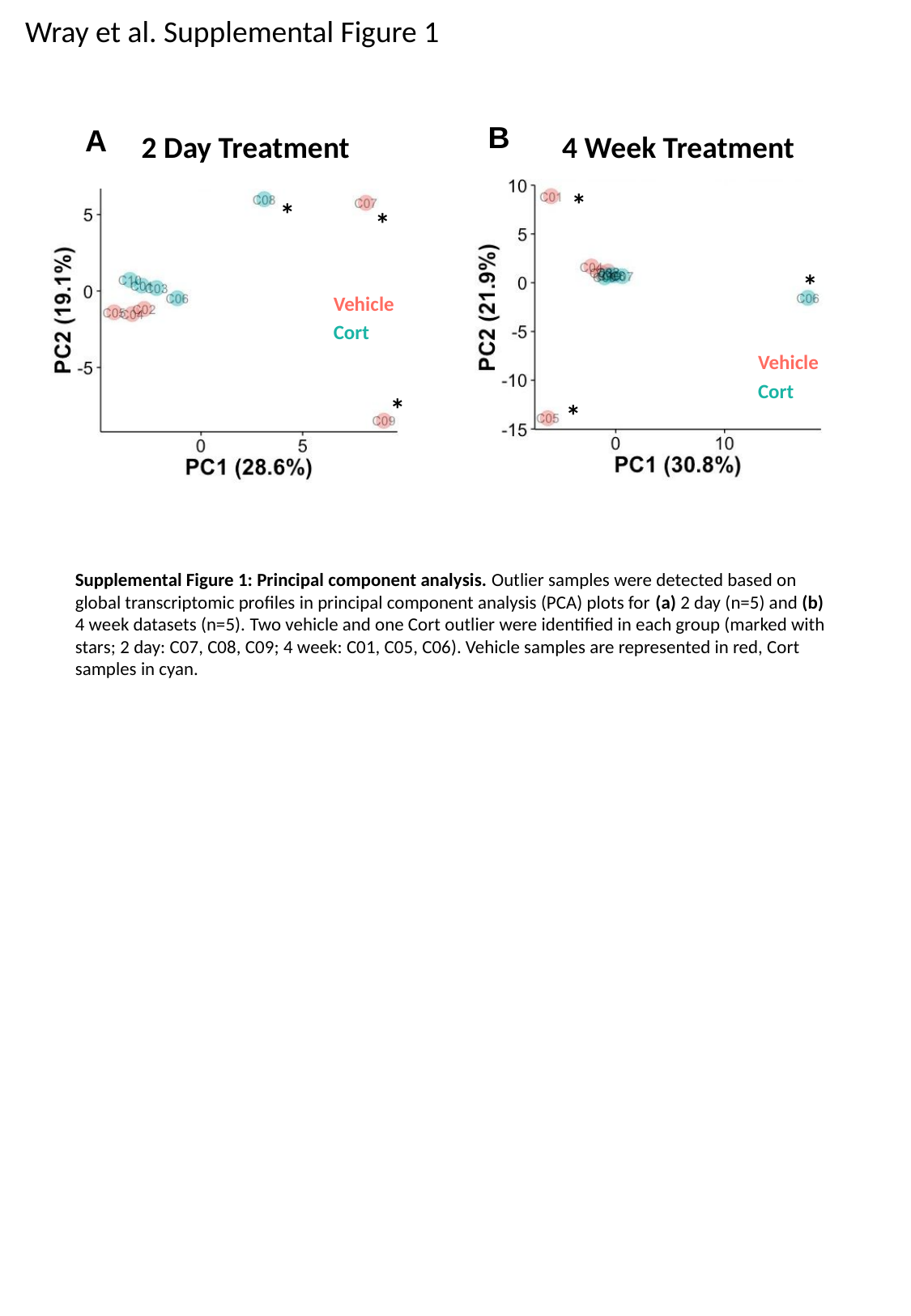

Wray et al. Supplemental Figure 1
B
A
2 Day Treatment
*
*
Vehicle
Cort
*
4 Week Treatment
*
*
Vehicle
Cort
*
Supplemental Figure 1: Principal component analysis. Outlier samples were detected based on global transcriptomic profiles in principal component analysis (PCA) plots for (a) 2 day (n=5) and (b) 4 week datasets (n=5). Two vehicle and one Cort outlier were identified in each group (marked with stars; 2 day: C07, C08, C09; 4 week: C01, C05, C06). Vehicle samples are represented in red, Cort samples in cyan.

## Slide 2
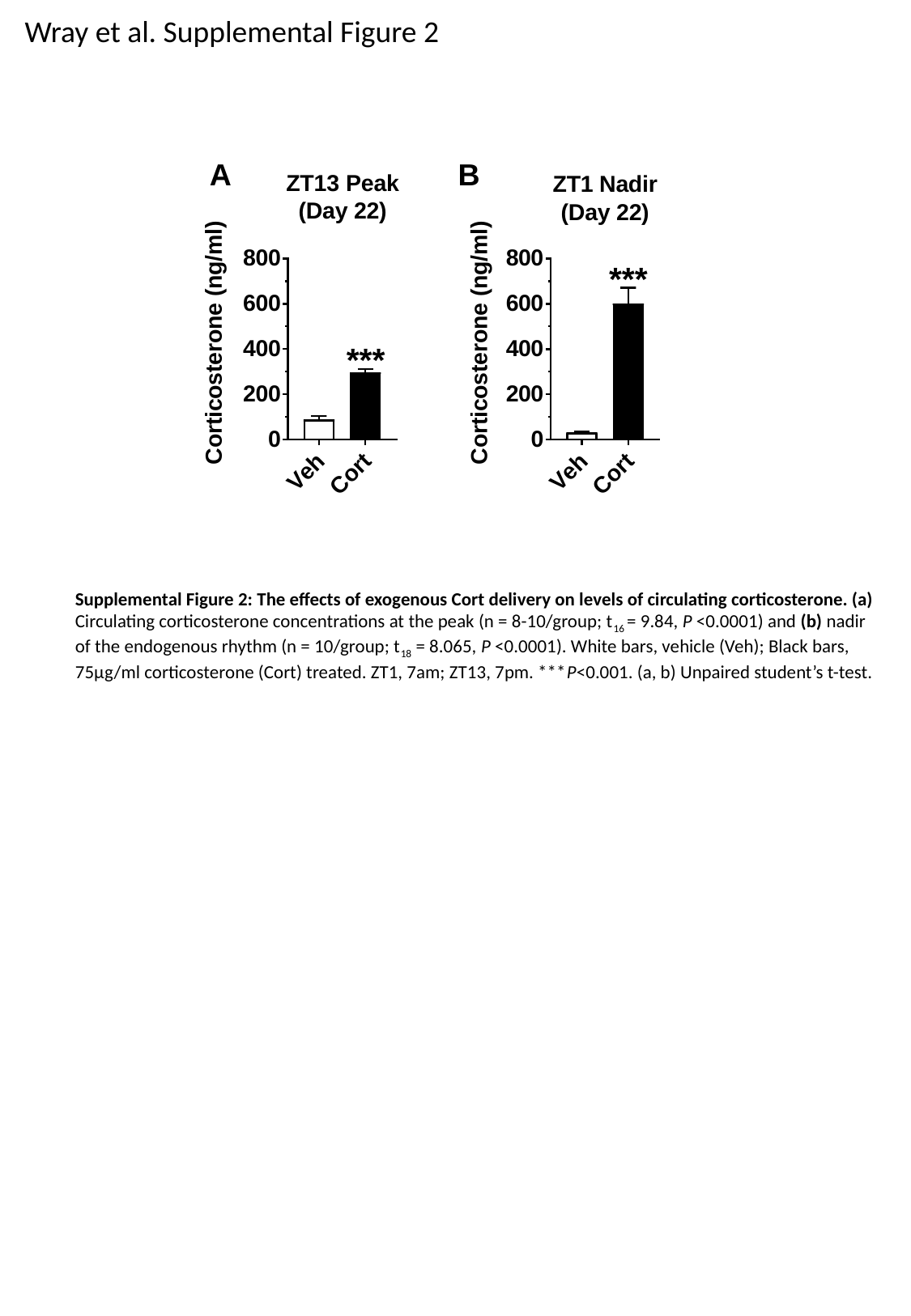

Wray et al. Supplemental Figure 2
A
B
Supplemental Figure 2: The effects of exogenous Cort delivery on levels of circulating corticosterone. (a) Circulating corticosterone concentrations at the peak (n = 8-10/group; t16 = 9.84, P <0.0001) and (b) nadir of the endogenous rhythm (n = 10/group; t18 = 8.065, P <0.0001). White bars, vehicle (Veh); Black bars, 75µg/ml corticosterone (Cort) treated. ZT1, 7am; ZT13, 7pm. ***P<0.001. (a, b) Unpaired student’s t-test.

## Slide 3
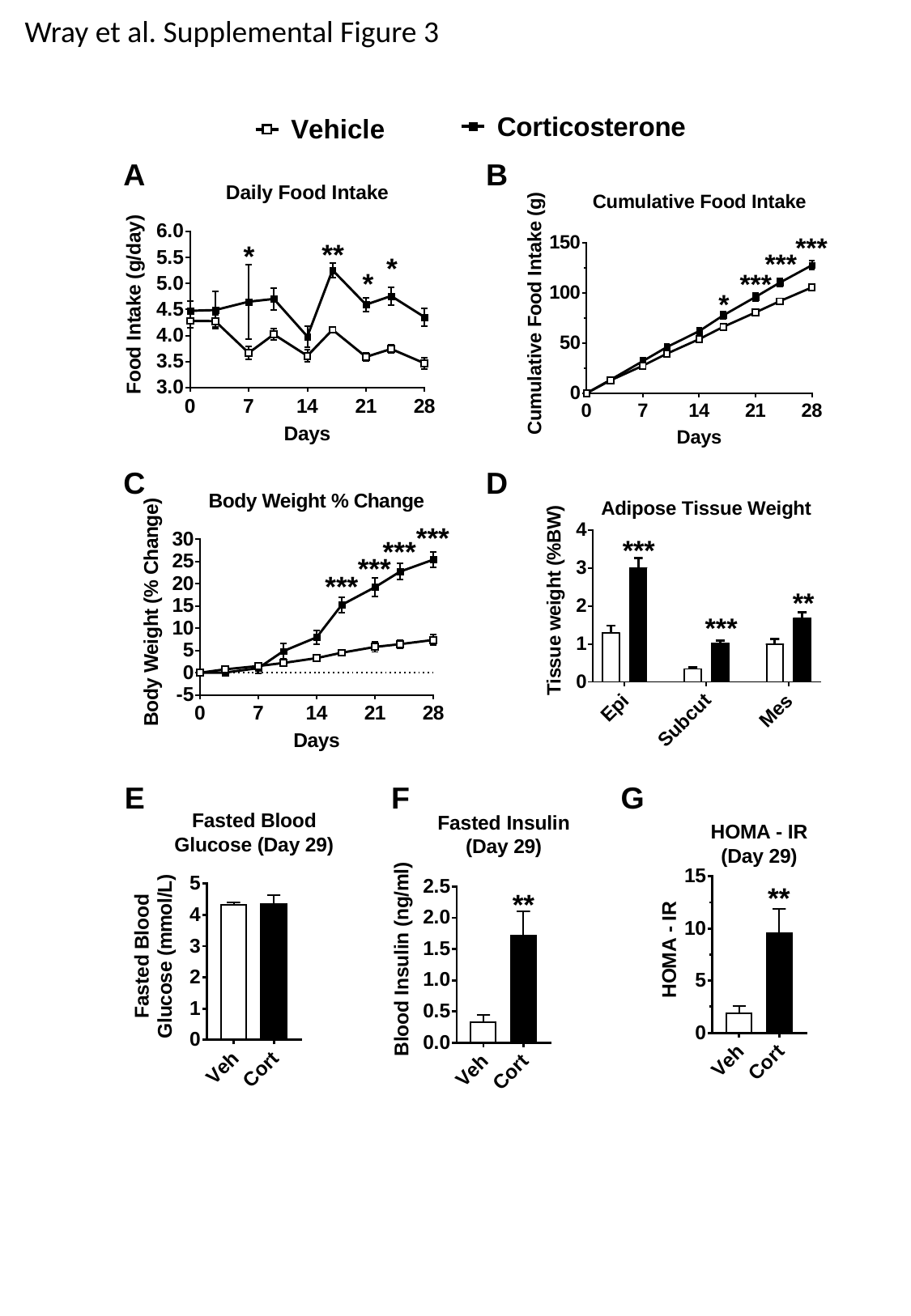

Wray et al. Supplemental Figure 3
A
B
C
D
E
F
G

## Slide 4
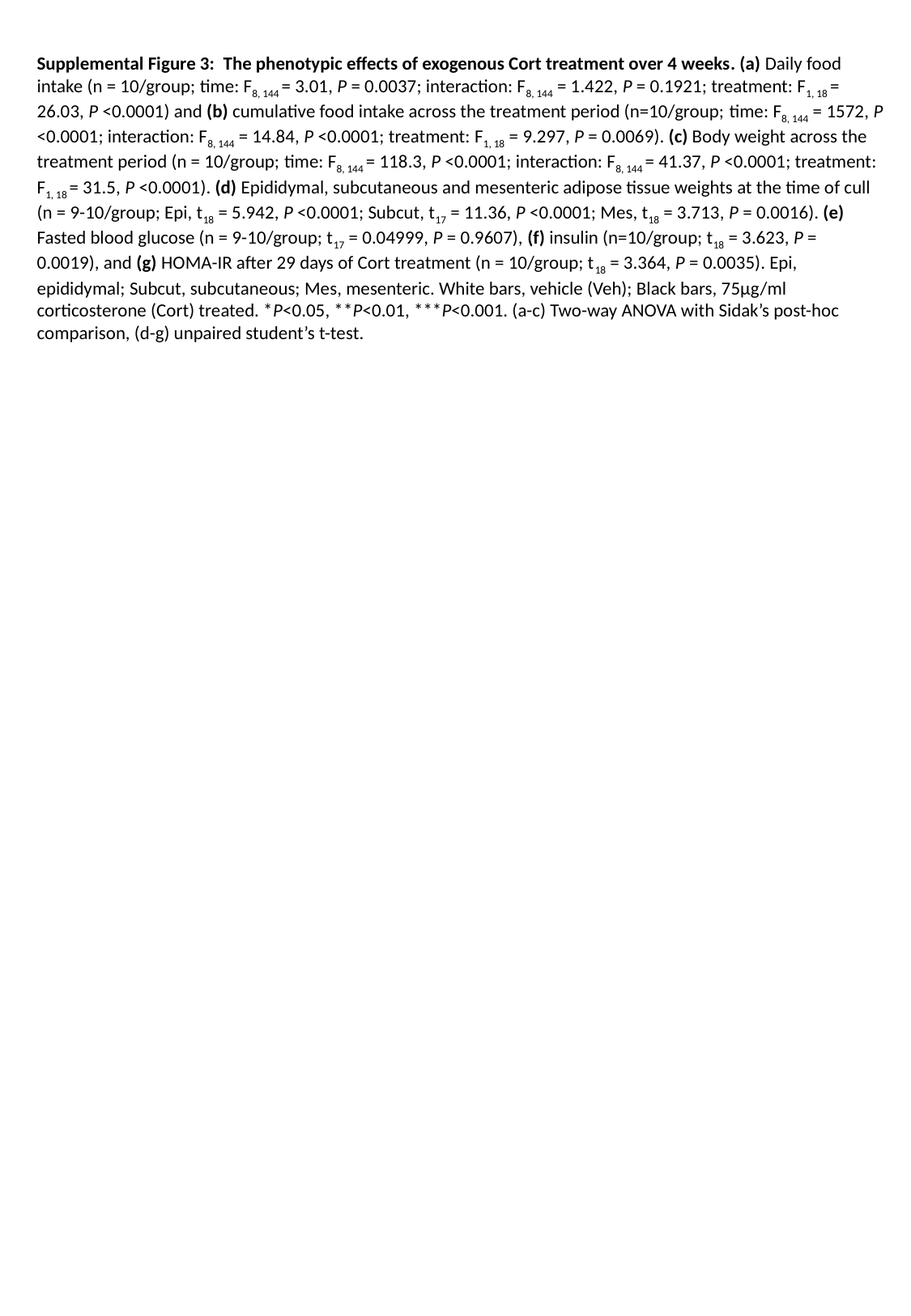

Supplemental Figure 3: The phenotypic effects of exogenous Cort treatment over 4 weeks. (a) Daily food intake (n = 10/group; time: F8, 144 = 3.01, P = 0.0037; interaction: F8, 144 = 1.422, P = 0.1921; treatment: F1, 18 = 26.03, P <0.0001) and (b) cumulative food intake across the treatment period (n=10/group; time: F8, 144 = 1572, P <0.0001; interaction: F8, 144 = 14.84, P <0.0001; treatment: F1, 18 = 9.297, P = 0.0069). (c) Body weight across the treatment period (n = 10/group; time: F8, 144 = 118.3, P <0.0001; interaction: F8, 144 = 41.37, P <0.0001; treatment: F1, 18 = 31.5, P <0.0001). (d) Epididymal, subcutaneous and mesenteric adipose tissue weights at the time of cull (n = 9-10/group; Epi, t18 = 5.942, P <0.0001; Subcut, t17 = 11.36, P <0.0001; Mes, t18 = 3.713, P = 0.0016). (e) Fasted blood glucose (n = 9-10/group; t17 = 0.04999, P = 0.9607), (f) insulin (n=10/group; t18 = 3.623, P = 0.0019), and (g) HOMA-IR after 29 days of Cort treatment (n = 10/group; t18 = 3.364, P = 0.0035). Epi, epididymal; Subcut, subcutaneous; Mes, mesenteric. White bars, vehicle (Veh); Black bars, 75µg/ml corticosterone (Cort) treated. *P<0.05, **P<0.01, ***P<0.001. (a-c) Two-way ANOVA with Sidak’s post-hoc comparison, (d-g) unpaired student’s t-test.

## Slide 5
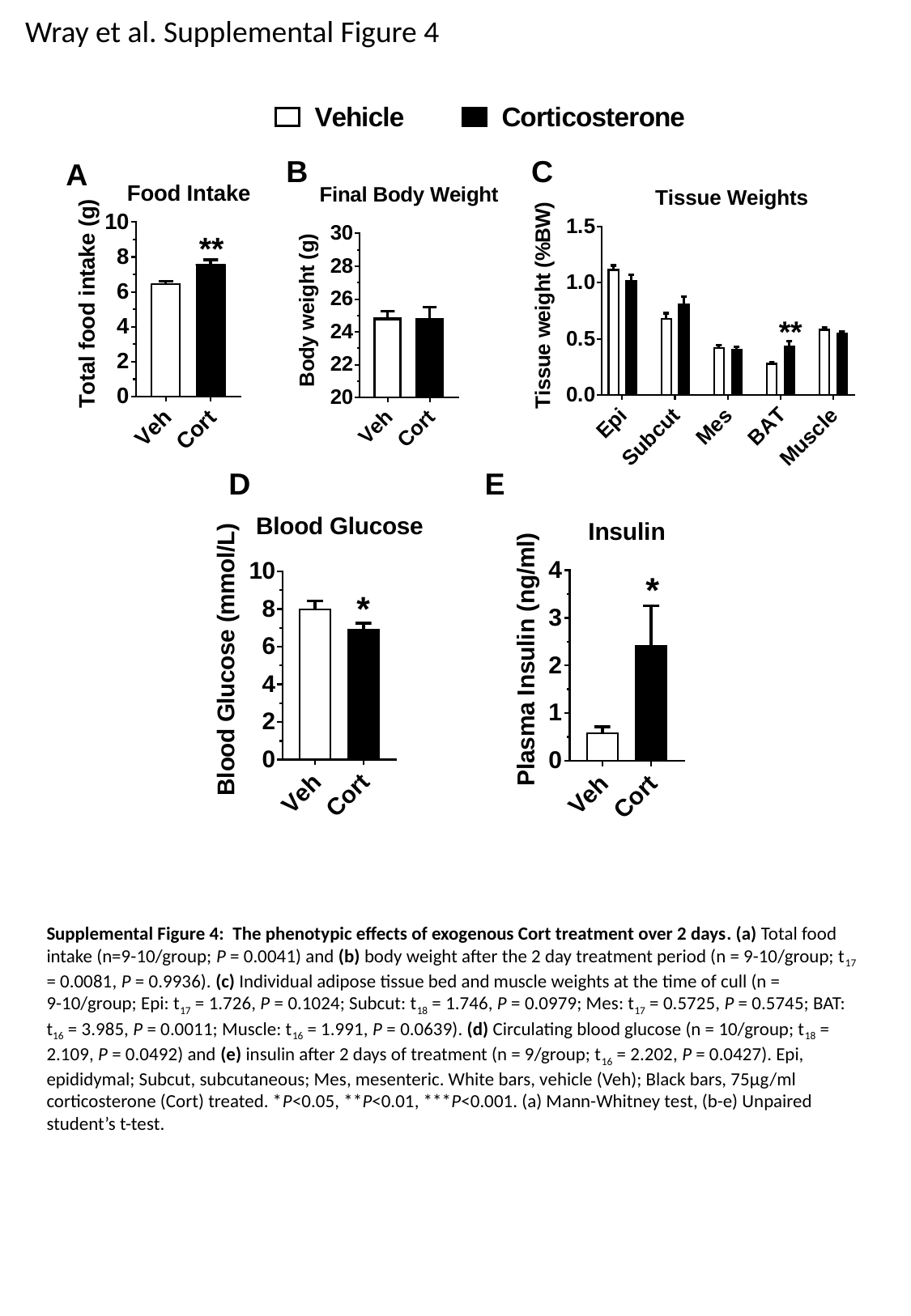

Wray et al. Supplemental Figure 4
B
C
A
D
E
Supplemental Figure 4: The phenotypic effects of exogenous Cort treatment over 2 days. (a) Total food intake (n=9-10/group; P = 0.0041) and (b) body weight after the 2 day treatment period (n = 9-10/group; t17 = 0.0081, P = 0.9936). (c) Individual adipose tissue bed and muscle weights at the time of cull (n = 9-10/group; Epi: t17 = 1.726, P = 0.1024; Subcut: t18 = 1.746, P = 0.0979; Mes: t17 = 0.5725, P = 0.5745; BAT: t16 = 3.985, P = 0.0011; Muscle: t16 = 1.991, P = 0.0639). (d) Circulating blood glucose (n = 10/group; t18 = 2.109, P = 0.0492) and (e) insulin after 2 days of treatment (n = 9/group; t16 = 2.202, P = 0.0427). Epi, epididymal; Subcut, subcutaneous; Mes, mesenteric. White bars, vehicle (Veh); Black bars, 75µg/ml corticosterone (Cort) treated. *P<0.05, **P<0.01, ***P<0.001. (a) Mann-Whitney test, (b-e) Unpaired student’s t-test.

## Slide 6
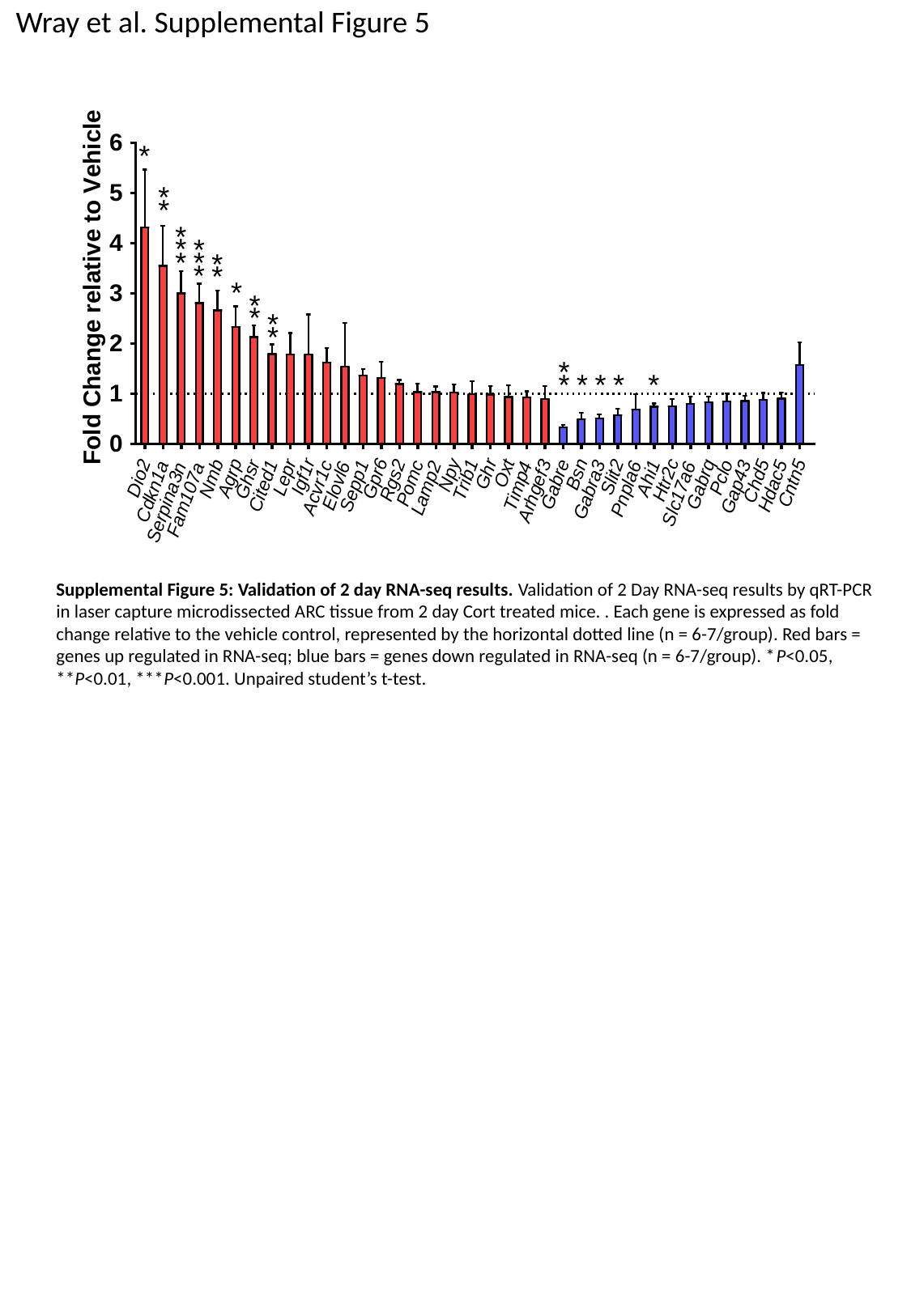

Wray et al. Supplemental Figure 5
Supplemental Figure 5: Validation of 2 day RNA-seq results. Validation of 2 Day RNA-seq results by qRT-PCR in laser capture microdissected ARC tissue from 2 day Cort treated mice. . Each gene is expressed as fold change relative to the vehicle control, represented by the horizontal dotted line (n = 6-7/group). Red bars = genes up regulated in RNA-seq; blue bars = genes down regulated in RNA-seq (n = 6-7/group). *P<0.05, **P<0.01, ***P<0.001. Unpaired student’s t-test.

## Slide 7
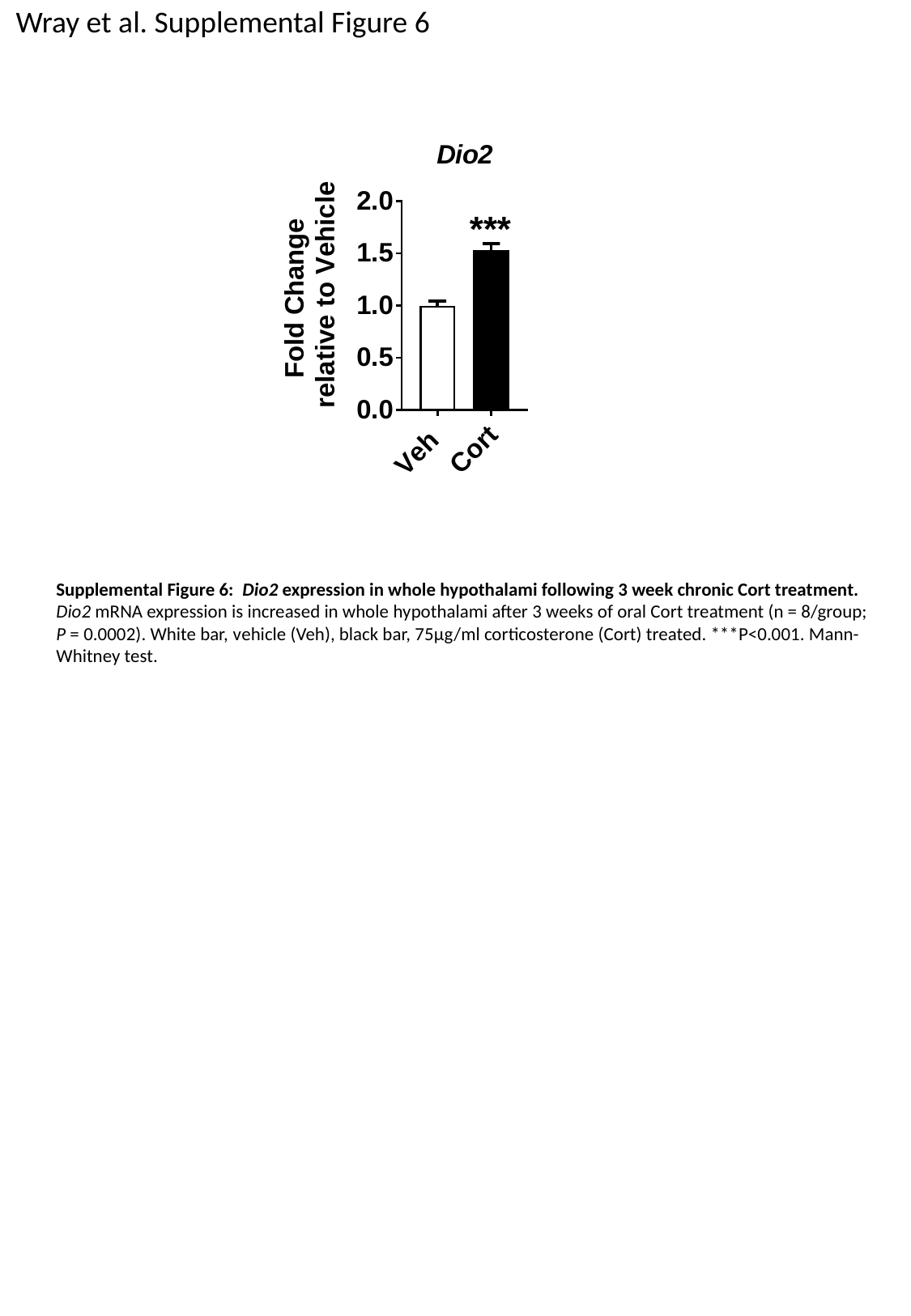

Wray et al. Supplemental Figure 6
Supplemental Figure 6: Dio2 expression in whole hypothalami following 3 week chronic Cort treatment. Dio2 mRNA expression is increased in whole hypothalami after 3 weeks of oral Cort treatment (n = 8/group; P = 0.0002). White bar, vehicle (Veh), black bar, 75µg/ml corticosterone (Cort) treated. ***P<0.001. Mann-Whitney test.

## Slide 8
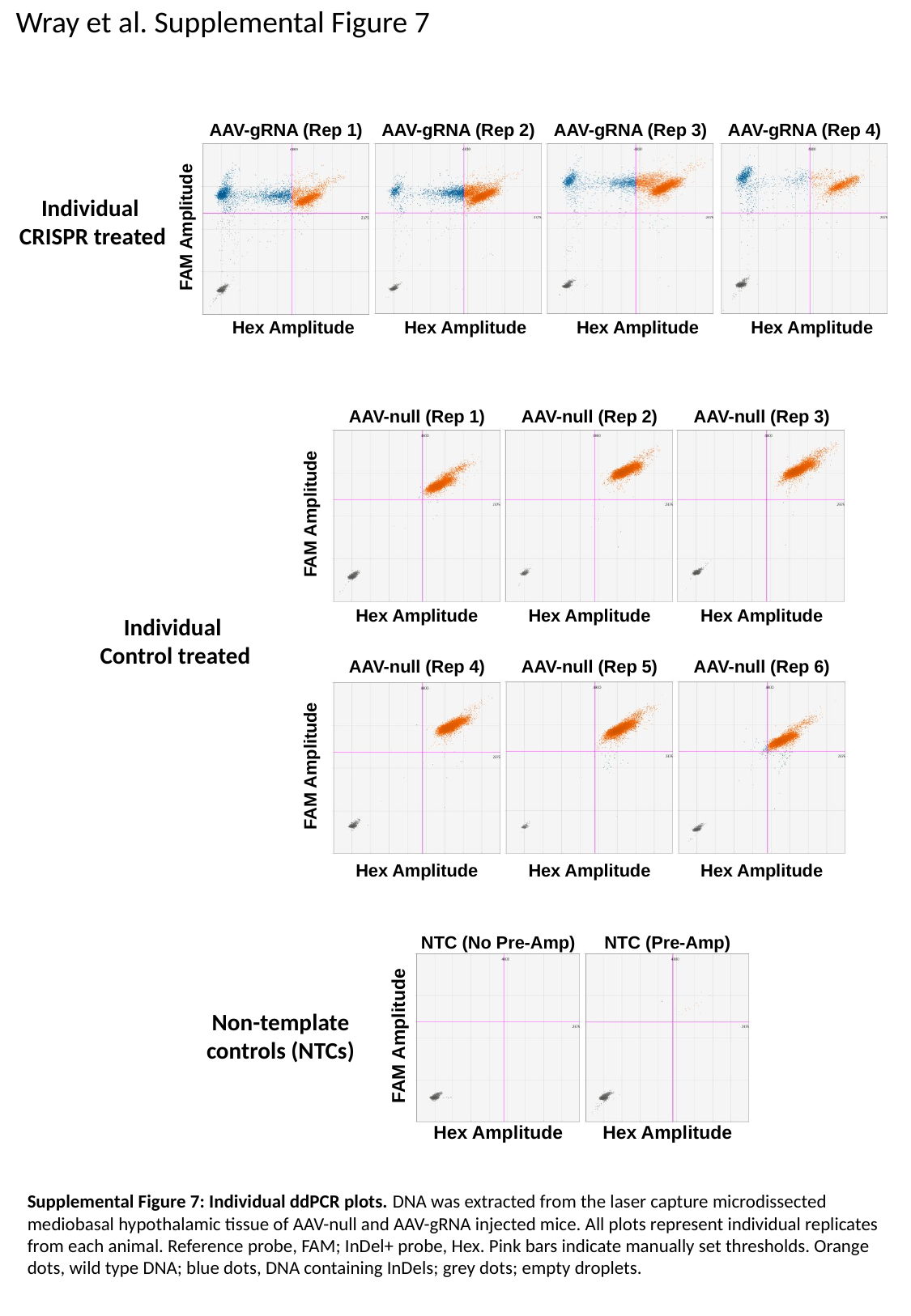

Wray et al. Supplemental Figure 7
AAV-gRNA (Rep 1)
AAV-gRNA (Rep 2)
AAV-gRNA (Rep 3)
AAV-gRNA (Rep 4)
FAM Amplitude
Hex Amplitude
Hex Amplitude
Hex Amplitude
Hex Amplitude
Individual
CRISPR treated
AAV-null (Rep 1)
AAV-null (Rep 2)
AAV-null (Rep 3)
FAM Amplitude
Hex Amplitude
Hex Amplitude
Hex Amplitude
AAV-null (Rep 4)
AAV-null (Rep 5)
AAV-null (Rep 6)
FAM Amplitude
Hex Amplitude
Hex Amplitude
Hex Amplitude
Individual
Control treated
NTC (No Pre-Amp)
NTC (Pre-Amp)
FAM Amplitude
Hex Amplitude
Hex Amplitude
Non-template controls (NTCs)
Supplemental Figure 7: Individual ddPCR plots. DNA was extracted from the laser capture microdissected mediobasal hypothalamic tissue of AAV-null and AAV-gRNA injected mice. All plots represent individual replicates from each animal. Reference probe, FAM; InDel+ probe, Hex. Pink bars indicate manually set thresholds. Orange dots, wild type DNA; blue dots, DNA containing InDels; grey dots; empty droplets.

## Slide 9
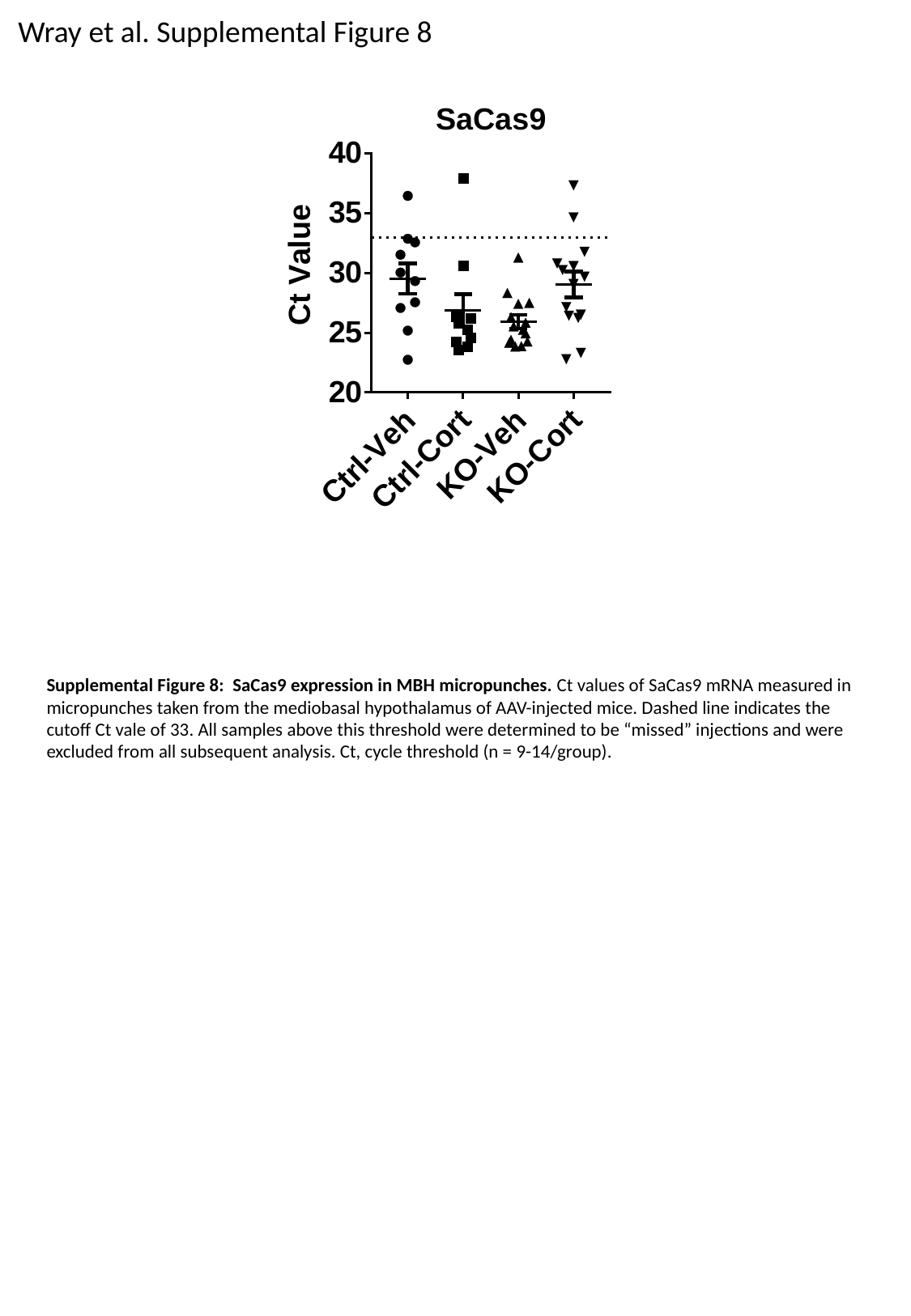

Wray et al. Supplemental Figure 8
Supplemental Figure 8: SaCas9 expression in MBH micropunches. Ct values of SaCas9 mRNA measured in micropunches taken from the mediobasal hypothalamus of AAV-injected mice. Dashed line indicates the cutoff Ct vale of 33. All samples above this threshold were determined to be “missed” injections and were excluded from all subsequent analysis. Ct, cycle threshold (n = 9-14/group).
